# Chloroplast phylogenomics and the taxonomy of *Saxifraga* section *Ciliatae* (Saxifragaceae)

**DOI:** 10.1002/ece3.9694

**Published:** 2023-01-06

**Authors:** Rui Yuan, Xiaolei Ma, Zhuoxin Zhang, Richard J. Gornall, Yongcui Wang, Shilong Chen, Qingbo Gao

**Affiliations:** ^1^ Key Laboratory of Adaptation and Evolution of Plateau Biota, Northwest Institute of Plateau Biology & Institute of Sanjiangyuan National Park Chinese Academy of Sciences Xining China; ^2^ University of Chinese Academy of Sciences Beijing China; ^3^ College of Forestry and Landscape Architecture South China Agricultual University Guanzhou China; ^4^ Department of Genetics University of Leicester Leicester UK; ^5^ Qinghai Provincial Key Laboratory of Crop Molecular Breeding Xining China

**Keywords:** chloroplast, phylogeny, plastome, *Saxifraga*, sect. *Ciliatae*

## Abstract

Comprising ca. 200 species, *Saxifraga* sect. *Ciliatae* is the most species‐rich section of *Saxifraga* s.str., whose center of diversity is in the Tibeto‐Himalayan region. The infra‐sectional classification of sect. *Ciliatae* is still in debate due to the high level of species richness, as well as remarkable variations of habitat, morphology, physiology and life cycles. Subdivisions of this section proposed in various taxonomic systems have not been adequately tested in previous phylogenetic studies, partly due to low taxonomic sampling density, but also to the use of few DNA markers. In order to achieve a more robust infra‐sectional classification of sect. *Ciliatae*, complete chloroplast genomes of 94 taxa from this section were analyzed, of which 93 were newly sequenced, assembled and annotated. The length of the 94 plastomes of sect. *Ciliatae* taxa range from 143,479 to 159,938 bp, encoding 75 to 79 unique protein‐coding genes (PCGs). Analyses of the 94 plastomes revealed high conservation in structural organization, gene arrangement, and gene content. Gene loss and changes of IR boundaries were detected but in extremely low frequency. The molecular phylogenetic tree from concatenated PCGs and complete chloroplast genome sequences exhibited high resolution and support values and confirms that sect. *Ciliatae* is monophyletic. Three well‐supported clades were revealed within the section that agree relatively well with the subsectional taxonomy of Gornall (1987), but some minor modifications should be made. Firstly, the monotypic subsection *Cinerascentes* should be abandoned and its constituent species, *S*. *cinerascens*, assigned to subsect. *Gemmiparae*. Secondly, subsections *Rosulares* and *Serpyllifoliae* should be merged and become subsect. *Rosulares*. Section *Ciliatae* thus comprises: subsect. *Hirculoideae* Engl. & Irmsch.; subsect. *Rosulares* Gornall; subsect. *Gemmiparae* Engl. & Irmsch.; subsect. *Flagellares* (C. B. Clarke) Engl. & Irmsch. and subsect. *Hemisphaericae* (Engl. & Irmsch.) Gornall.

## Graphical abstract

Subdivisions of sect. *Ciliatae* proposed in various taxonomic systems have not been adequately tested in previous phylogenetic studies, partly due to low taxonomic sampling density, but also to the use of few DNA markers. In this study, based on the 94 chloroplast genomes of sect. *Ciliatae*, we explored the phylogenetic relationships of this group.

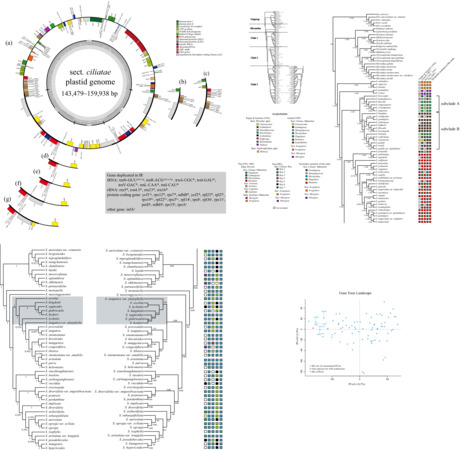

## 1 INTRODUCTION

Comprising ca. 450–500 species (Pan et al., 2001; Webb & Gornall, 1989), *Saxifraga* L. is the largest genus of Saxifragaceae s.str. (Soltis, 2007). It is found in mountainous regions of the northern hemisphere, primarily in Eurasia, but with a few circumpolar species, and some extending southwards down the Rocky Mountain/Andean Cordillera (Ebersbach, Muellner‐Riehl, et al., 2017; Gao et al., 2015; Tkach et al., 2015). Partly owing to its great morphological variability, the genus has been of great interest in evolutionary studies (e.g. Abbott & Comes, 2003; Dechaine et al., 2013; Ebersbach, Muellner‐Riehl, et al., 2017; Ebersbach, Schnitzler, et al., 2017; Ebersbach et al., 2018; Gao et al., 2015; Healy & Gillespie, 2004; Tkach et al., 2015; Zhang et al., 2008). The traditional circumscription of *Saxifraga* by Engler and Irmscher (1916/1919), Gornall (1987) and Soltis et al. (1996) has been shown to be polyphyletic, with *Saxifraga* sect. *Micranthes* (Haw.) D. Don and sect. *Merkianae* (Engl. & Irmsch.) Gornall being more closely related to *Chrysosplenium* L. They are best recognized as a separate genus, *Micranthes* Haw. (e.g. Deng et al., 2015; Soltis et al., 2013; Tkach et al., 2015; Xiang et al., 2012). *Saxifraga* s.s. is sister to all other genera in the family (Xiang et al., 2012).

A recent phylogenetic study of ca. 50% of *Saxifraga* species employed sequence data of the internal transcribed spacer (ITS) region of nuclear ribosomal DNA (nrDNA) and the *trn*L‐*trn*F region of plastid DNA (Tkach et al., 2015). The authors recognized at least 13 sections. Among them, sect. *Ciliatae* Haw. is the largest, comprising over 200 species, nearly half the genus, and is the focus of the present study. The section has a center of diversity in the Tibeto‐Himalayan region (THR), which includes the Qinghai‐Tibetan Plateau, the Himalayas, and the Hengduan Mountains (Mosbrugger et al., 2018). Species from the section inhabit rock faces, scree slopes, tundra, alpine meadows and woodland margins, a range of habitats similar to those occupied by species outside the section in Europe. Not surprisingly, this habitat diversity is reflected by extensive morphological, physiological and life cycle diversity. For example, habit ranges from cushions or spreading mats to tall, single‐stemmed herbs; some species produce filiform stolons; some are obligate calcicoles or calcifuges; and, whilst most are perennial, some are monocarpic perennial or even annual species.

The first authors to attempt an infra‐sectional classification of sect. *Ciliatae* in any detail were Engler and Irmscher (1912a, 1912b), who arranged what was then called sect. *Hirculus* (Haw.) Tausch. (= sect. *Ciliatae*) into 11 groups based on resemblances in gross morphology. Initially, the 11 taxa were described at subsections, although they were also referred to as greges (Engler & Irmscher, 1912a); a few months later all mention of subsections had been dropped and they were exclusively designated as greges (Engler & Irmscher, 1912b). The latter treatment was reprised in a formal monograph of the whole genus (Engler & Irmscher, 1916/1919). It is clear that it was difficult to assess the relationships among the 11 greges, as is evident from the scheme proposed by Engler and Irmscher (1912b), in which grex *Hirculoideae* was regarded as central and to which the other 10 greges were more or less equally related (Figure S1). Gornall (1987) revised Engler and Irmscher's (1912b, 1916/1919), classification by recognizing the greges *Hirculoideae*, *Gemmiparae*, *Cinerascentes*, *Flagellares* and *Hemisphaericae* as subsections, as was originally proposed (Engler & Irmscher, 1912a); by subsuming greges *Stellariifoliae*, *Turfosae* and *Densifoliatae* into grex *Hirculoideae* (converted to series rank) and recognizing greges *Nutantes* and *Lychnitidae* as series, all within subsect. *Hirculoideae*; and by splitting grex *Sediformes* into two subsections: *Rosulares* and *Serpyllifoliae*, based on whether or not a prominent basal leaf‐rosette is developed. In total, Gornall (1987) recognized seven subsections and six series, although no species lists were given. Shortly afterwards, in an account of the genus in China, Pan (1991, 1992) adopted a novel approach to the classification of the species by focusing heavily on ovary position and petal morphology, in which the number of calloses and the number of veins were deemed important characters. He distributed the species across the three sections included within his subgenus *Hirculus* (Haw.) Torr. & Gray, viz. *Ciliatae*, *Ligulatae* and *Aretiaria*. Within his section *Ciliatae* he recognized four subsections, 16 series and four subseries. Within his section *Ligulatae*, he recognized three subsections and 13 series, including taxa that all other botanists assign to sects. *Bronchialis* and *Porphyrion*. His third section, *Aretiaria*, was not subdivided further. Leaving aside a number of nomenclatural issues, the resulting system was revised significantly by Pan et al. (2001), and only a single section was recognized, sect. *Ciliatae*, consistent with the treatment of Gornall (1987). Although the ranks of subsection and series were not used, species were accommodated in informal subgroups corresponding to seven identification keys in which leaf venation and the nature and distribution of hairs featured prominently.

Molecular phylogenetic studies have been applied to the classification of sect. *Ciliatae*. Zhang et al. (2008) pointed out that sect. *Ciliatae* as delimited by Engler and Irmscher (1916/1919) and Gornall (1987) was monophyletic, based on ITS sequences. This result was subsequently confirmed by Gao et al. (2015) using the combined dataset of *psbA*‐*trn*H, *trn*L‐*trn*F and ITS sequences, and three major clades were revealed: clade 1 contains species belonging to *Ciliatae* subsects. *Gemmiparae* Engl. & Irmsch., *Cinerascentes* Engl. & Irmsch., *Flagellares* Gornall, and *Hemisphaericae* (Engl. & Irmsch.) Gornall; clade 2 contains species belonging to *S*. subsects. *Rosulares* Gornall and *Serpyllifoliae* Gornall; and clade 3 corresponds to the largest group in section *Ciliatae*, viz. subsect. *Hirculoideae*. However, no formal taxonomic proposals were made owing to: (i) low sampling density (27 *Ciliatae* species in Zhang et al., 2008; 42 in Gao et al., 2015; 71 in Tkach et al., 2015); and (ii) low to moderate support for some lineages because of employing only a few DNA markers. In all such studies the contents of each of the three subclades were largely unresolved.

In this paper, we aim to improve matters by using whole chloroplast (cp) genomes. Chloroplast genomes are now widely employed in phylogenetic studies, due to their conservative character in terms of structure, gene type and gene order (Gao et al., 2019). Analysis of complete cp genomes has the advantage of the potential to significantly improve the resolution of phylogenetic relationships in large, complex plant lineages (Jansen et al., 2007), even in enigmatic taxa (Dong et al., 2018). In this study, we combined a high sampling density with whole cp genome sequences to: (i) reconstruct a robust phylogeny of sect. *Ciliatae*; and (ii) use the phylogeny to test earlier taxonomic systems and propose revisions where necessary.

## 2 MATERIALS AND METHODS

### 2.1 Taxon sampling

Since sect. *Ciliatae* as delimited by Engler and Irmscher (1916/1919) and Gornall (1987) has been shown to be monophyletic (Gao et al., 2015; Tkach et al., 2015; Zhang et al., 2008), and our main aim of this study is to address infra‐sectional classification, our sampling strategy focused on taxa within sect. *Ciliatae*. A total of 99 taxa of *Saxifraga* are included, of which 94 are from sect. *Ciliatae*, comprising ca. 50% of the species diversity of this section (Table S1). These species represent: (i) eight of the 11 greges of Engler and Irmscher (1916/1919); (ii) all seven subsections of Gornall (1987); (iii) all three sections and six of the seven subsections of Pan (1991, 1992); and (iv) species from all seven keys of Pan et al. (2001). As outgroups, we included 17 representatives from other genera of Saxifragaceae as well as more distantly related taxa from Grossulariaceae and Iteaceae. This resulted in a final data matrix of 122 complete cp genome sequences, of which 103 were newly generated in this study (93 taxa from sect. *Ciliatae*; two from sect. *Mesogyne* Sternb. and *Irregulares* Haw., respectively; and six from the genus *Micranthes*). Leaf material was collected in the field and dried in silica gel. The taxon, locality, voucher information and Genbank accession numbers are listed in Table S1. Voucher specimens are deposited in the herbarium of Northwest Institute of Plateau Biology (HNWP), Xining, Qinghai, China.

### 2.2 Sequencing and quality control

Total genomic DNA was extracted from silica‐dried leaves by the DNA quick extraction system (DP321) according to the manufacturer's protocol (Tiangen Biochemical Technology Co., Ltd.). Total DNA was then randomly fragmented with the Covaris ultrasonic crusher. A series of steps were performed to complete library construction, such as end repair and phosphorylation, a‐tail addition, sequencing connector addition, purification, and PCR amplification. Finally, the qualified libraries were pooled into flowcells. NovaSeq 6000 (Illumina Inc.) was used for sequencing after cBOTs clustering, with paired‐end methods (150 bp). We used fastp v.0.23.1 (Chen et al., 2018) to filter raw sequence reads when: (i) the *N* content in any read was more than 10% of the base; (ii) the number of low quality (*Q* ≤ 5) bases in any read exceeded 50%; and (iii) any read contained the adapter content; if so, the paired reads were removed (Yan et al., 2013).

### 2.3 Chloroplast genome assembly, annotation, and genome structure analysis

High‐quality clean reads were assembled using GetOrganelle v.1.7.5 (Jin et al., 2020) with the default parameters and *S. sinomontana* J‐T. Pan & Gornall as a reference genome (GenBank accession no. MN104589; Li et al., 2019). Complete plastomes were flexibly annotated in batches using PGA v.3 (Altschul et al., 1990; Qu et al., 2019). The preliminarily annotation of complete plastid genomes downloaded from GenBank may have some problems, such as format errors and a reversed SSC area, we therefore re‐annotated them also with PGA. The correctness of the gene products was then manually checked for common errors, e.g. missing start or stop codons, and interrupted translation products. Finally, the modified sequence was submitted to ORGDRAW's online tool for chloroplast genome visualization (Lohse et al., 2007).

We employed a comprehensive bioinformatics tool, PhyloSuite v.1.2.2 (Zhang, Gao, et al., 2020), to calculate GC content of the 94 sect. *Ciliatae* plastomes included in this study. Simple sequence repeats (SSRs) were identified using the MISA‐web program (Beier et al., 2017), using the threshold of 10 repeat units for mono‐, 6 for di‐, 4 for tri‐, 3 for tetra‐, penta‐ and hexanucleotide SSRs. The mVISTA program (Frazer et al., 2004) was used to compare the 94 *S*. sect. *Ciliatae* plastid genomes under a Shuffle‐LAGAN model. Nucleotide variability (Pi) was calculated using DnaSP v.6 (Rozas et al., 2017), and highly variable sites among sect. *Ciliatae* plastomes were discovered with a step size of 100 bp and window length of 25 bp. CodonW v.1.4.4 (http://codonw.sourceforge.net/) was employed to obtain the amount of codon encoded by chloroplast genes and the relative synonymous codon usage (RSCU). RSCU was the ratio of a codon's frequency of use to its expected frequency, with a value greater than one indicating that the codon was biased. To calculate the ratio of nonsynonymous to synonymous rates (d*N*/d*S*), we used the CODEML option with model 0 (same d*N*/d*S* ratios for each branch) as implemented in PAML v.4.9 (Yang, 2007). To do so, 74 common protein‐coding genes (PCGs) were divided into 16 groups of gene families shared by the 94 sect. *Ciliatae* plastomes after removing the too short gene of *psbF*. The 16 gene groups were *accD*, *atp* (*atpA*, *atpB*, *atpE*, *atpF*, *atpH*, *atpI*), *ccsA*, *cemA*, *clpP*, *infA, matK*, *ndh* (*ndhB ndhC*, *ndhD*, *ndhE*, *ndhF*, *ndhH*, *ndhJ*, *ndhK*), *pet* (*petA*, *petB*, *petD, petG*, *petL*, *petN*), *psa* (*psaA*, *psaB*, *psaC*, *psaI*, *psaJ*), *psb* (*psbA*, *psbB*, *psbC*, *psbD*, *psbE*, *psbH*, *psbI*, *psbJ*, *psbK*, *psbL*, *psbM*, *psbN, psbT*, *psbZ*), *rbcL*, *rpl* (*rpl2*, *rpl14*, *rpl16*, *rpl20*, *rpl22*, *rpl23*, *rpl32*, *rpl33*, *rpl36*), *rpo* (*rpoA*, *rpoB*, *rpoC1*, *rpoC2*), *rps* (*rps2*, *rps3*, *rps4*, *rps7*, *rps8*, *rps11*, *rps12*, *rps15*, *rps16*, *rps18*, *rps19*), *ycf* (*ycf1*, *ycf2*, *ycf3*, *ycf4*). Finally, the d*N*/d*S* difference between above‐mentioned functional gene groups was calculated.

### 2.4 Phylogenetic analyses

Two data matrices, the whole plastid sequences and PCGs, were employed to conduct phylogenetic analyses. As for the data matrix of PCGs, we extracted 81 PCGs shared by all 122 taxa employed in this study after removing the following genes: *infA*_copy2, *ndhA*, *ndhG*, *ndhH*_copy2, *ndhI*, *petD*_copy2, *rpl2*_copy3, *rpl14*_copy2, *rpl22*_copy2, *rpl36*_copy2, *rpoA*_copy2, *rps3*_copy2, *rps8*_copy2, *rps11*_copy2, *rps14*, *rps15*_copy2, *rps19*_copy2, *ycf1*_copy2. Multiple sequences were originally aligned using MAFFT v.7.313 (Katoh & Standley, 2013), and “auto” strategy was used to match the optimal algorithm for different sequence types. In order to optimize the results, the aligned sequences were then trimmed in trimAl v.1.2 (Capella‐Gutiérrez et al., 2009) to ensure a uniform ending. The program Gblocks v.0.91b (Talavera & Castresana, 2007) was used to remove the poorly aligned positions and divergent regions from the multi‐sequence alignment results. Furthermore, individual PCGs were refined in batch using MACSE v.2 (Ranwez et al., 2018). Individual aligned, trimmed and refined PCGs were then concatenated into a single matrix for the following phylogenetic analyses. Phylogenetic reconstructions were conducted by means of maximum likelihood (ML) and Bayesian inference (BI) as implemented in PhyloSuite (Zhang, Gao, et al., 2020). Substitution models for the ML and BI analyses were chosen on the basis of the Akaike information criterion (AIC) using ModelFinder as implemented in IQ‐TREE v.1.6.12 (Kalyaanamoorthy et al., 2017; Nguyen et al., 2015). The best fitting model was GTR + F + I + G4 for the ML and BI analyses for both data matrix of complete plastid sequences and PCGs. The ML analyses were performed using IQ‐TREE with 1000 bootstrap replicates. The BI analyses were conducted using MrBayes v.3.2.6 by running for 10 million generations with four parallel Markov Chains Monte Carlo (MCMC; Ronquist et al., 2012). Samples were taken every 1000 generations and the first 25% trees were discarded as burn‐in. Phylogenetic trees were visualized and embellished using the online tool ITOL v.6 (Letunic & Bork, 2007).

### 2.5 Performance test of individual PCGs to species trees

To investigate heterogeneity among gene trees, sequences of each PCG were used to reconstruct individual gene trees by mean of ML. The gene trees were then combined in ASTRAL v.5.7.8 to form a species tree with coalescence (Mirarab et al., 2014). In addition, the phylogenetic tree derived from ML based on concatenated PCGs, as mentioned above, was also employed to conduct the heterogeneity test. In total, the dataset consisted of 79 PCG trees, a gene species tree and a phylogenetic tree of the 122 taxa. The R package TREESPACE v.1.1.4.1 (Jombart et al., 2017; Zhang, Sun, et al., 2020) was employed to calculate the pairwise distances within and between gene trees and species trees, and to plot the statistical distribution of the trees by the unrooted Robinson‐Foulds algorithm, following the framework of Gonçalves et al. (2019). Since TREESPACE only accepts groups of trees with the same tips, we removed the *psaJ* and *accD* locus from PCGs dataset. Finally, we conducted a principal coordinate analysis (PCoA) to analyze any inconsistency between gene trees and species trees.

## 3 RESULTS

### 3.1 Plastome structure of sect. *Ciliatae*

**FIGURE 1:**
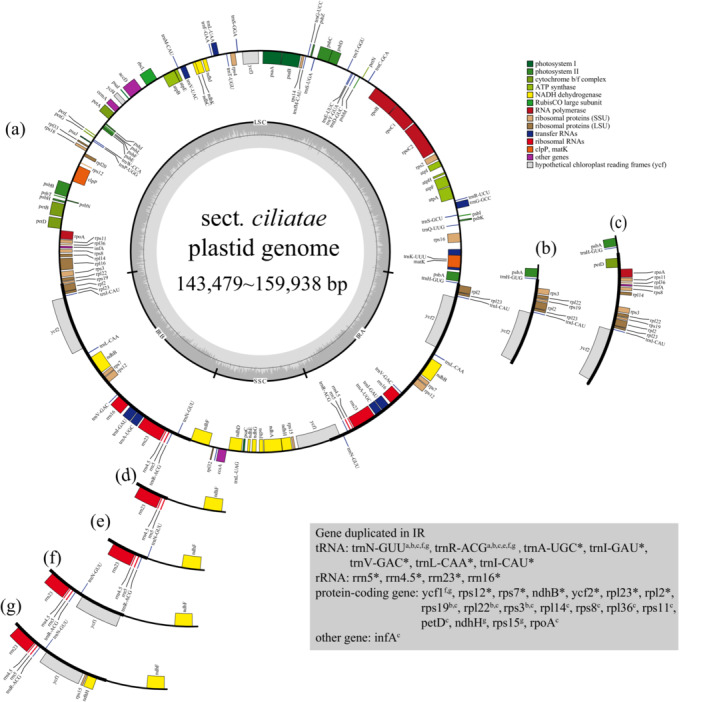
Plastid structure of *S*. sect. *Ciliatae*, a‐f represent different structural types, which are divided according to the difference of IR region. Genes located in the IR region are shown in the gray box at the bottom right.

The length of assembled plastomes of the 94 sect*. Ciliatae* taxa ranged from 143,479 bp (*S. brunneopunctata* H. Sm.) to 159,938 bp (*S. insolens* Irmsch.), encoding 75 to 79 unique PCGs (Figure 1; Table 1). All the plastomes present a typical circular, quadripartite structure, i.e. a large single‐copy (LSC) region (72,478–81,585 bp), a small single‐copy (SSC) region (13,189–27,592 bp), and two inverted repeat (IR) regions (22,921–3526 bp; Table 1). The GC contents are particularly similar, ranging from 37.8% in *S. brunonis* Wall. ex Ser. and *S. cinerascens* Engl. & Irmsch. to 38.4% in *S. diversifolia* Wall. ex Ser. (Table 1). Since the expansion and contraction of the IR region is the main cause of variation in plastome size, we classified sect*. Ciliatae* plastomes into seven types (a–g) based on the changes in IR boundary (Figure 1). All the 94 sect. *Ciliatae* plastomes correspond to type a, except for six which were classified as types b–g, viz. *S. consanguinea* W.W. Sm. (type b); *S. aristulata* J.D. Hook. & Thoms. (type c); *S. montanella* H. Sm. (type d); *S. diversifolia* (type e); *S. insolens* (type f); and *S. moorcroftiana* (Wall. ex Ser.) Sternb. (type g). The majority of sect. *Ciliatae* plastomes involved in this study contain 11 *ndh* genes, namely *ndhA*‐*ndhK*, which encode a protein complex that catalyzes the transfer of electrons from NADH to plastoquinone at photosystem I (Martín & Sabater, 2010). Loss of *ndh* genes happens in only one of the 94 representatives of sect. *Ciliatae*, namely *S. brunneopunctata*, in which *ndhA*, *ndhG* and *ndhI* are lost. Another gene loss event occurs in *S. nigroglandulifera* Balakr., in which *rps14* is missing.

**TABLE 1:** Characteristics of the sect. *Ciliatae* plastomes generated in this study

Taxon	LSC length/bp	SSC length/bp	IR length/bp	Genome size/bp	GC content/%	Genome type	Gene duplicated in IR (protein‐coding/tRNA/rRNA/other gene)
*S. angustata*	81,478	16,634	24,915	147,942	38	a	6/7/4/0
*S. aristulata*	72,478	16,860	33,526	156,390	37.9	c	15/7/4/1
*S. aristulata var. longipila*	79,772	16,891	25,507	147,677	38.1	a	6/7/4/0
*S. atuntsiensis*	80,115	16,650	25,338	147,441	38.1	a	6/7/4/0
*S. aurantiac*	80,057	16,514	25,296	147,163	38	a	6/7/4/0
*S. auriculata*	79,739	16,865	25,478	147,560	38.1	a	6/7/4/0
*S. auriculata var. conaensis*	79,990	16,884	25,542	147,958	38.1	a	6/7/4/0
*S. balfourii*	80,789	16,818	25,685	148,977	38	a	6/7/4/0
*S. bergenioides*	80,218	16,960	25,452	148,082	37.9	a	6/7/4/0
*S. brachypoda*	80,850	16,788	25,699	149,036	37.9	a	6/7/4/0
*S. brevicaulis*	81,256	16,892	25,650	149,448	38	a	6/7/4/0
*S. brunneopunctata*	79,770	13,189	25,260	143,479	38.2	a	6/7/4/0
*S. brunonis*	81,585	16,758	25,731	149,805	37.8	a	6/7/4/0
*S. chumbiensis*	79,897	16,954	25,495	147,841	38	a	6/7/4/0
*S. cinerascens*	81,581	16,902	25,631	149,745	37.8	a	6/7/4/0
*S. congestiflora*	79,623	16,898	25,524	147,569	38.1	a	6/7/4/0
*S. consanguinea*	78,877	16,658	27,214	149,963	37.8	b	9/7/4/0
*S. diversifolia*	79,766	24,746	23,815	152,142	38.4	e	6/6/4/0
*S. diversifolia var. angustibracteata*	79,745	16,901	25,353	147,352	38.1	a	6/7/4/0
*S. drabiformis*	79,980	16,492	25,422	147,316	38	a	6/7/4/0
*S. eglandulosa*	79,815	16,742	27,681	151,919	38.1	a	6/7/4/0
*S. egregia*	79,581	16,846	25,479	147,385	38.1	a	6/7/4/0
*S. egregia var. eciliata*	79,730	16,865	25,479	147,553	38.1	a	6/7/4/0
*S. erectisepala*	79,701	16,881	25,387	147,356	38.1	a	6/7/4/0
*S. filicaulis*	80,850	16,788	25,699	149,036	37.9	a	6/7/4/0
*S. flaccida*	80,945	16,779	25,497	148,718	38	a	6/7/4/0
*S. gemmigera*	79,796	16,681	25,333	147,143	38.1	a	6/7/4/0
*S. gemmigera var. gemmuligera*	79,797	16,681	25,333	147,144	38.1	a	6/7/4/0
*S. gemmipara*	81,444	16,758	25,689	149,580	37.9	a	6/7/4/0
*S. glabricaulis*	79,512	16,865	25,497	147,371	38.1	a	6/7/4/0
*S. glacialis*	79,888	16,637	25,263	147,051	38	a	6/7/4/0
*S. gouldii*	81,307	16,913	25,587	149,394	37.9	a	6/7/4/0
*S. gouldii var. eglandulosa*	81,222	16,900	25,589	149,300	37.9	a	6/7/4/0
*S. gyalana*	79,980	16,693	25,246	147,165	38	a	6/7/4/0
*S. heleonastes*	79,676	16,810	25,539	147,564	38	a	6/7/4/0
*S. hemisphaerica*	80,861	16,913	25,718	149,210	37.9	a	6/7/4/0
*S. hirculoides*	79,853	16,866	25,453	147,625	38	a	6/7/4/0
*S. hispidula*	80,710	16,828	25,506	148,550	38	a	6/7/4/0
*S. hookeri*	79,474	16,938	25,437	147,286	38.1	a	6/7/4/0
*S. hypericoides*	79,821	16,935	25,482	147,720	38.1	a	6/7/4/0
*S. implicans*	79,674	23,578	24,840	152,932	38.2	a	6/7/4/0
*S. insolens*	79,781	14,981	32,588	159,938	37.9	f	7/7/4/0
*S. isophylla*	79,823	16,879	25,478	147,658	38.1	a	6/7/4/0
*S. kingdonii*	79,736	16,914	25,460	147,570	38.1	a	6/7/4/0
*S. lepida*	80,075	18,090	25,269	148,703	38	a	6/7/4/0
*S. litangensis*	79,858	16,913	25,441	147,653	38.1	a	6/7/4/0
*S. lychnitis*	79,615	16,901	25,421	147,358	38.1	a	6/7/4/0
*S. maxionggouensis*	79,537	22,217	24,827	151,408	38.2	a	6/7/4/0
*S. montanella*	79,603	27,592	23,045	153,285	38.2	d	6/5/4/0
*S. moorcroftiana*	79,860	19,035	29,912	158,719	38.1	g	9/7/4/0
*S. nanella*	80,030	16,534	25,431	147,426	38	a	6/7/4/0
*S. nangqenica*	79,809	16,914	25,518	147,759	37.9	a	6/7/4/0
*S. nangxianensis*	81,173	16,748	24,877	147,675	37.9	a	6/7/4/0
*S. nigroglandulifera*	80,044	16,975	25,518	148,055	37.9	a	6/7/4/0
*S. oresbia*	79,587	16,802	25,462	147,313	38.1	a	6/7/4/0
*S. pardanthina*	79,744	16,870	25,348	147,310	38.1	a	6/7/4/0
*S. parnassiifolia*	79,983	16,762	25,626	147,997	37.9	a	6/7/4/0
*S. parva*	79,665	16,909	25,506	147,586	38	a	6/7/4/0
*S. perpusilla*	79,979	16,540	25,452	147,423	38	a	6/7/4/0
*S. pratensis*	79,778	16,881	25,348	147,355	38.1	a	6/7/4/0
*S. przewalskii*	79,628	16,963	25,516	147,623	37.9	a	6/7/4/0
*S. pseudohirculus*	79,707	16,859	25,478	147,522	38.1	a	6/7/4/0
*S. punctulata*	79,715	16,522	25,417	147,071	38	a	6/7/4/0
*S. saginoides*	79,676	16,842	25,487	147,492	38.1	a	6/7/4/0
*S. sanguinea*	79,446	16,442	25,456	146,800	38	a	6/7/4/0
*S. sediformis*	79,693	16,545	25,444	147,126	37.9	a	6/7/4/0
*S. signata*	79,889	16,727	25,228	147,072	37.9	a	6/7/4/0
*S. signatella*	79,489	16,327	25,373	146,562	38.1	a	6/7/4/0
*S. sikkimensis*	79,925	16,817	27,787	152,316	38.1	a	6/7/4/0
*S. sinomontana*	79,310	16,874	25,528	147,240	38	a	6/7/4/0
*S. sinomontana var. amabilis*	79,782	16,856	25,522	147,682	38	a	6/7/4/0
*S. stella‐aurea*	80,007	16,534	25,431	147,403	38.1	a	6/7/4/0
*S. stellariifolia*	79,798	16,866	25,478	147,620	38.1	a	6/7/4/0
*S. subaequifoliata*	79,711	21,843	22,921	147,396	38.1	a	6/7/4/0
*S. substrigosa*	80,761	17,026	25,480	148,747	38	a	6/7/4/0
*S. tangutica*	79,723	16,923	25,525	147,696	38	a	6/7/4/0
*S. tangutica var. platyphylla*	79,709	16,867	25,494	147,564	38	a	6/7/4/0
*S. taraktophylla*	80,005	16,678	25,282	147,247	38.1	a	6/7/4/0
*S. tibetica*	79,970	16,875	25,530	147,905	37.9	a	6/7/4/0
*S. tsangchanensis*	80,011	16,687	25,783	148,264	38	a	6/7/4/0
*S. umbellulata*	79,224	16,389	25,382	146,377	38.1	a	6/7/4/0
*S. umbellulata var. pectinata*	79,939	16,518	25,468	147,393	38	a	6/7/4/0
*S. unguiculata*	79,963	16,512	25,488	147,451	38.1	a	6/7/4/0
*S. unguiculata var. limprichtii*	80,066	16,520	25,296	147,178	38.1	a	6/7/4/0
*S. unguiculata var. subglabra*	79,962	16,704	25,345	147,356	38.1	a	6/7/4/0
*S. uninervia*	79,967	16,670	25,273	147,183	38.1	a	6/7/4/0
*S. vilmoriniana*	79,383	16,533	25,457	146,830	38	a	6/7/4/0
*S. viridipetala*	81,476	16,756	25,684	149,600	38	a	6/7/4/0
*S. viscidula*	79,873	16,938	25,392	147,595	38	a	6/7/4/0
*S. wallichiana*	81,475	16,877	25,533	149,418	37.9	a	6/7/4/0
*S. wardii*	81,182	16,904	25,589	149,264	37.9	a	6/7/4/0
*S. xiaozhongdianensis*	79,878	16,804	25,490	147,662	38.1	a	6/7/4/0
*S. yarlungzangboensis*	79,664	16,925	25,392	147,373	38	a	6/7/4/0
*S. yushuensis*	79,566	16,491	25,433	146,923	38	a	6/7/4/0

The total number of SSRs identified in the 94 sect*. Ciliatae* plastomes ranged from 49 (*S. heleonastes* H. Sm., *S. saginoides* J.D. Hook. & Thoms.) to 82 (*S. angustata* H. Sm.; Table S2). We detected six different repeat patterns, ranging from mononucleotide (p1) to hexanucleotide (p6). The results showed that p1 is most abundant, accounting for a proportion of 65.85%–85.45%, while pentanucleotide (p5) and p6 occurred with relatively low frequency (0%–5.8%, 0%–7.32%, respectively). A further count of the number of A/T and G/C repeats in p1 showed that the frequency of A/T repeats (37–58) was much higher than that of G/C (1–4; Figure S2). Highly divergent regions among the 94 sect*. Ciliatae* plastomes were assessed using mVISTA with the annotation of *S. sinomontana* as the reference genome. Pi values were then calculated using a sliding window method in DnaSP to further detect highly variable sites among sect. *Ciliatae* plastomes. The results showed that coding regions of *matK*, *rpoC2*, *ycf1*, *ndhE*, *ndhD* and *ccsA* were highly divergent among PCGs, whereas, the most divergent noncoding regions were detected as intergenic spacers of *petA*‐*psbJ*, *trnK‐UUU*_*rps16*, *rps16*‐*trnQ*_*UUG*, *trnT*‐*UGU*_*trnL‐UAA, trnL‐UAA*_*trnF‐GAA*, *ndhF*_*rpl32*, *rpl32*_*trnL‐UAG* (Figures S3 and S4). In the codon preference analysis, the total number of codons detected among the 94 sect*. Ciliatae* plastomes ranged from 47,826 (*S. brunneopunctata*) to 53,312 (*S. insolens*), representing 64 codon types. Arginine (Arg) encoded by AGA had the highest RSCU value (1.90) of the 64 codon types, while Arg encoded by CGC had the lowest (RSCU = 0.48; Figure S5). Among the 64 codon types, 34 are preferred codons (RSCU > 1), 2 are non‐preferred codons (RSCU = 1), and 28 are low‐usage codons (RSCU < 1). Most of the preferred codons in the study are ended by A or U, however, the two codons that were identified as non‐preferred are due to the uniqueness for coding Methionine and Tryptophan, respectively. The d*N*/d*S* values of all genes are less than 1, with median values ranging from 0.024 of *pet* to 0.565 of *ccsA* for the 16 functional gene groups. *Ycf1* (d*N*/d*S* = 0.709) and *ycf2* (d*N*/d*S* = 0.731) in *ycf* group, as well as *psbK* (d*N*/d*S* = 0.7325) in *psb* group exhibit the highest d*N*/d*S* values (Figure S6).

### 3.2 Phylogenetic analysis

**FIGURE 2:**
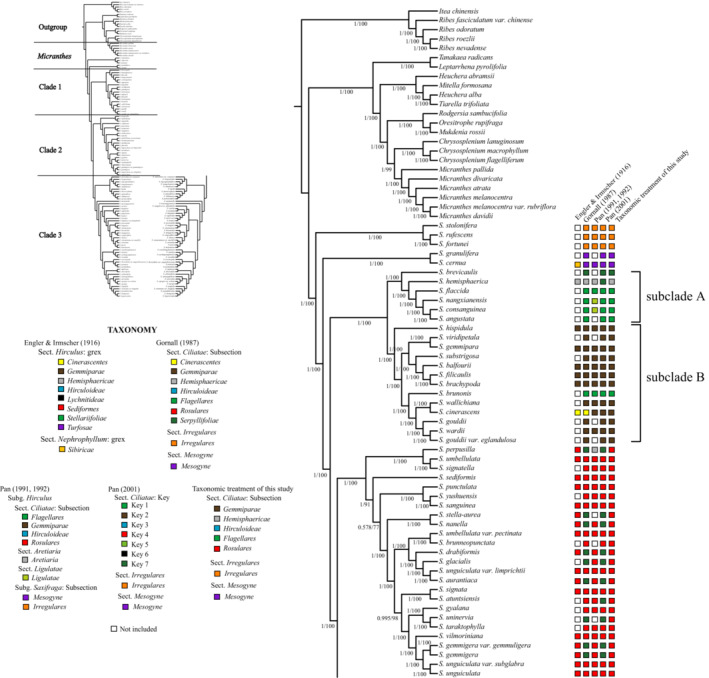
Phylogenetic tree reconstructed based on the full genome sequence using Bayesian interference (BI) and maximum likelihood (ML) methods. Taxa include *S*. sect. *Ciliatae*, *Micranthes*, and outgroups in this study, but except *S*. subsect. *Hirculoideae*. Numbers at the nodes represent BI posterior probability (PP) and ML bootstrap (BS) values greater than 50%. The taxonomic information of *S*. sect. *Ciliatae* in previous studies and this study is arranged in columns.

**FIGURE 3:**
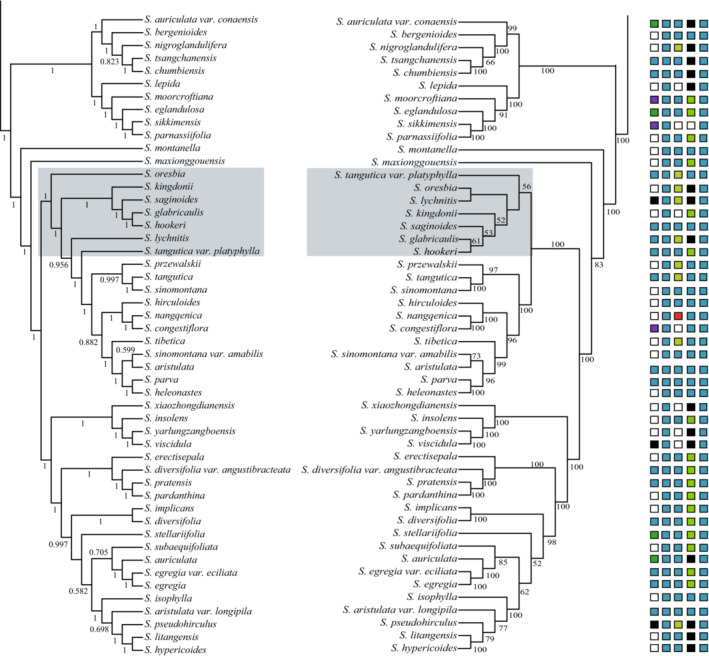
The figure is followed by Figure 3. Based on the full genome sequence from *S*. subsect. *Hirculoideae*, the phylogenetic tree was reconstructed by Bayesian interference (left) and maximum likelihood (right) methods. Numbers at the nodes represent BI posterior probability (PP) and ML bootstrap (BS) values greater than 50%. The taxonomic information of *S*. subsect. *Hirculoideae* is arranged in columns according to right tree. The legend of taxonomy is shown in Figure 2.

Four phylogenetic trees were reconstructed by means of ML and BI, based on the complete chloroplast genome sequences and 81 shared PCGs. Topologies of the four phylogenetic trees were similar, except for some minor inconsistencies in subsect. *Hirculoideae* (Figures 2 and 3; Figure S7). Phylogenetic trees of sect. *Ciliatae* as reconstructed from complete chloroplast genome sequences were more robust than those recovered from PCG sequences. All representatives of sect*. Ciliatae* included in this study form a monophyletic lineage with high support values (Bootstrap support, BS = 100%, posterior probability PP = 1.00; Figures 2 and 3; Figure S7). Within sect. *Ciliatae*, three well‐supported clades are recognized as also revealed by Gao et al. (2015), but with generally higher resolution and support values, as well as minor position changes of tips within each clade. We follow Gao et al. (2015) in naming these clades and describe the phylogenetic results in the context of the taxonomy of Gornall (1987).

Clade 1 (BS = 100%, PP = 1.00) comprises species of subsects. *Hemisphaericae*, *Flagellares*, *Gemmiparae* and *Cinerascentes*, as well as *S. brevicaulis* H. Sm., a species that had been provisionally allocated to subsect. *Serpyllifoliae*. Two well supported subclades (both with BS = 100%, PP = 1.00) are identified within clade 1. In the first subclade, *S. brevicaulis* occupies a basal position, followed in turn by *S. hemisphaerica* J.D. Hook. & Thoms. (subsect. *Hemisphaericae*, BS = 100%, PP = 1.00) and then a well‐supported monophyletic group of four species of subsect. *Flagellares* (*S. flaccida* J‐T. Pan, *S. nangxianensis* J‐T. Pan, *S. consanguinea*, *S. angustata*; BS = 100%, PP = 1.00). The second subclade comprises all representatives of subsect. *Gemmiparae*, and in which *S. brunonis* (subsect. *Flagellares*) and *S. cinerascens* (subsect. *Cinerascentes*) are embedded.

Clade 2 (BS = 100%, PP = 1.00) consists of all species of subsect. *Rosulares* and subsect. *Serpyllifoliae* included in this study. These subsections are not recovered as monophyletic groups.

Clade 3 (BS = 100, PP = 100) contains all representatives belonging to subsect. *Hirculoideae*. Three major subclades are recognized within this species‐rich group. Notably, the positions of *S. oresbia* Anth., *S. lychnitis* J.D. Hook. & Thoms. and *S. tangutica* Engl. var. *platyphylla* (H. Sm.) J. T. Pan are inconsistent when comparing trees produced by complete chloroplast genome sequences with those from PCG sequences.

### 3.3 Performance of individual PCGs to species trees

**FIGURE 4:**
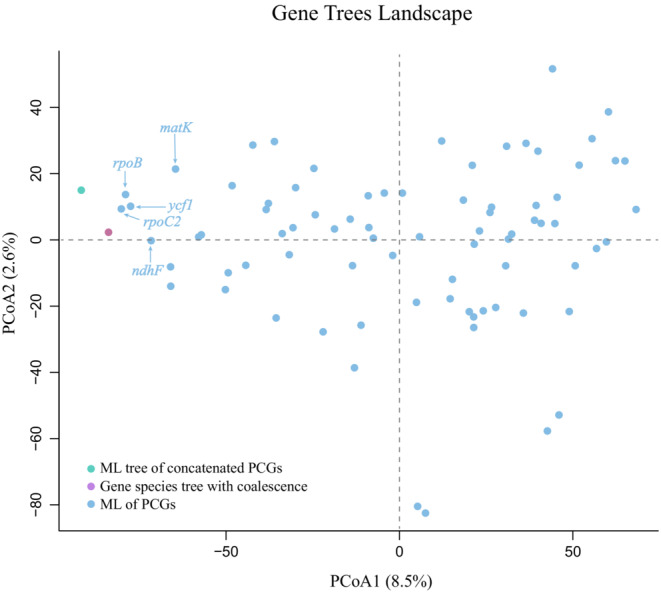
Discordance of plastid gene trees. Principal coordinate analysis depicting ordinations of five species trees versus 79 plastid protein‐coding gene (PCG) trees using unrooted Robinson‐Foulds algorithms. Since TREESPACE only accepts groups of trees containing the same tips, *psaJ* and *accD* locus was removed. The two species trees and gene trees based on *rpoB*, *rpoC2*, *ndhF*, *matK* and *ycf1*are indicated in the plots.

We employed PCoA to investigate the heterogeneity of gene trees, synthetic gene species tree with coalescence, and phylogenetic tree estimated based on ML of concatenated PCGs. The results revealed that the two species trees are distributed relatively close to each other, whereas individual gene trees exhibit great variation (Figure 4). The first and second axes of the PCoA explained 8.5% and 2.6% of the variation in tree topologies, respectively. The gene trees using *rpoB*, *rpoC2*, *ndhF*, *matK* and *ycf1* are close to gene species trees, of which the latter two are commonly used for phylogenetic studies. We provided the gene species tree with coalescence and gene trees based on *rpoB*, *rpoC2*, *ndhF*, *matK* and *ycf1* in Figure S8.

## 4 DISCUSSION

### 4.1 Plastome structure of sect. *Ciliatae*

In previous studies related to sect. *Ciliatae*, whole chloroplast genome data of only one species has been published (Li et al., 2019). In this study, chloroplast genomes of 103 taxa of Saxifragaceae were newly sequenced and assembled, of which 93 are from sect. *Ciliatae*. Structure analyses of these newly generated plastomes from sect. *Ciliatae* plus *S. sinomontana* from NCBI (National Center for Biotechnology Information; accession no. MN104589) show high conservation in structural organization, gene arrangement and gene content. Large structural variations, such as the deletion of IR regions as detected in *Pisum sativum* L., *Glycyrrhiza uralensis* Fisch., and *Cicer arietinum* L. (Duan et al., 2021), inversion of large fragments as have been frequently revealed in *Artemisia* L. (Liu et al., 2013), even gene transposition as in *Selaginella uncinata* (Desv.) Spring (Tsuji et al., 2007), are not detected in plastomes of sect. *Ciliatae*. However, gene loss was detected in plastomes of sect. *Ciliatae*, but with extremely low frequency. Gene loss, especially of *ndh* genes, has been detected in increasing number of higher plants including species of Orchidaceae (e.g., Chang et al., 2006; Lin et al., 2015; Niu et al., 2017; Yang et al., 2013), *Najas flexilis* (Willd.) Rostk. & Schmidt (Peredo et al., 2013), *Carnegiea gigantean* (Engelm.) Britton & Rose (Sanderson et al., 2015), and species of *Gentiana* L. (Sun et al., 2018). However, this seems not to be the case in plastomes of sect. *Ciliatae*. Loss of *ndh* genes happens only occurred in two species of this study, *S. brunneopunctata* and *S. nigroglandulifera* Balakr. The former is the loss of *ndhA*, *ndhG* and *ndhI*, as well as *rps14* of the latter. The *ndh* gene loss in various angiosperm groups could be an example of convergent evolution in plants (Sun et al., 2018). Considering that gene loss in plastomes is an ongoing process in evolution (Martin et al., 1998), the extremely low frequency of gene loss in *S*. sect. *Ciliatae* may reflect a relatively short evolutionary history of this species‐rich section, which has been confirmed by previous studies (Ebersbach, Muellner‐Riehl, et al., 2017; Ebersbach, Schnitzler, et al., 2017; Gao et al., 2015).

The boundaries of IRs and LSC/SSC differ in angiosperms (Raman et al., 2017), and expansion/contraction of the IR regions often lead to size variation of chloroplast genomes (Wang et al., 2008). In sect. *Ciliatae*, although 88 out of 94 taxa share similar IR boundaries, six species exhibit expansion/contraction of IRs, leading to duplication/loss of certain genes which ultimately result in size variation. In summary, although gene loss and size variation are detected, chloroplast genomes are rather conservative among species of sect. *Ciliatae*. Besides, highly divergent regions as revealed among plastomes of sect. *Ciliatae* are similar to that as revealed at the family level of Saxifragaceae (Li et al., 2019), and the range of SSRs numbers and frequency of different SSRs types are comparable with those in *S*. *sinomontana* (Li et al., 2019).

### 4.2 Performance of individual PCGs to species trees

Five gene trees based on *rpoB*, *rpoC2*, *ndhF*, *matK* and *ycf1*, show the closer to the two species trees in sect. *Ciliatae*. Among these PCGs, *matK* gene encodes the only mature enzyme related to chloroplast group II intron splicing in land plants. It is one of the most rapidly evolving genes and is widely used in phylogenetic reconstruction in angiosperms (Fan et al., 2022; Kim et al., 2019; Kuo et al., 2011; Wei et al., 2021). *Ycf1* is a coding region containing long tandem repeats. In many plant plastid genomes, the *ycf1* gene has undergone widespread insertions of the tandem repeat, leading to extreme length variation. These characteristics make this gene one of the most valuable markers in phylogenetic studies (Duan et al., 2020; Khandelwal et al., 2022; Li et al., 2021). The sequences of the five genes are relatively long, which can provide more effective resolution in phylogenetic analyses. It is worth noting that the phylogenetic inconsistency of individual gene trees is serious, emphasizing the importance of taking gene tree heterogeneity into account in phylogenetic studies.

### 4.3 Phylogenetic and taxonomic inferences in sect. *Ciliatae*

A total of 94 taxa of sect. *Ciliatae* are included in this study, representing 83 species and 11 varieties. This species sampling consists of about half of the species of sect. *Ciliatae* and represents nearly all subtaxa as proposed by Engler and Irmscher (1916/1919), Gornall (1987), Pan (1991, 1992) and Pan et al. (2001). It is thus the most complete molecular phylogenetic investigation of sect. *Ciliatae* carried out so far, especially at the level of plastomes. As has been shown in previous studies (Gao et al., 2015; Tkach et al., 2015; Zhang et al., 2008), sect. *Ciliatae* as delimited by Engler and Irmscher (1916/1919), Gornall (1987) and Pan et al. (2001), is a monophyletic group. The three main clades within sect. *Ciliatae* as revealed by this study are similar to those recovered by Gao et al. (2015) and Tkach et al. (2015), but with higher support values and much better resolution within each clade. The three major clades of sect. *Ciliata*e correspond reasonably well to subtaxa postulated in previous taxonomic systems based on morphological characters (Engler & Irmscher, 1912a, 1912b; Gornall, 1987; Pan et al., 2001), though with some exceptions. Based on our relatively large number of sampled taxa and comparatively well‐resolved and supported phylogenetic tree, some taxonomic issues can be addressed.

#### 4.3.1 Clade 1

Clade 1 comprises species from subsects. *Hemisphaericae*, *Flagellares*, *Gemmiparae* and *Cinerascentes*, as well as *S. brevicaulis* H. Sm. It is not clear how this clade as a whole can be recognized morphologically. It is divided into two well‐supported subclades. The first of these, subclade A, comprises basal taxa that are successively sister to a monophyletic group of species characterized by filiform stolons. The basal taxa include *S. brevicaulis* and *S. hemisphearica*, to which may be added *S. eschscholtzii* Sternb. (Tkach et al., 2015). The latter two species formed subsect. *Hemisphaericae* **(**Engler & Irmscher, 1912a, 1912b), characterized in part by hyaline, fimbriate leaf apical margins. Pan (1991, 1992) added *S. zhidoensis* J‐T. Pan and *S. perpusilla* J.D. Hook. & Thoms to this group. DNA sequence data shows that *S. zhidoensis* may also be one of the basal taxa since morphologically it is very similar in all respects to *S. hemisphearica*. In Gao et al. (2015) it clustered with the sister group of stoloniferous species, although support was not very strong and sampling was poor. The placement of at least *S*. *hemisphaerica* and *S. zhidoensis* alongside species from subsect. *Serpyllifoliae* (key 7 in Pan et al. (2001)) is not supported by our data, since all representatives of subsect. *Serpyllifoliae* belong with species of subsect. *Rosulares* to form a well‐supported clade (clade 2; BS = 100%, PP = 1.00). In contrast, sequence data from this study and from Tkach et al. (2015) shows that *S. perpusilla* belongs squarely in clade 2, a group comprising sections *Serpyllifoliae* and *Rosulares*.

The inclusion of *S. brevicaulis* H.Sm. among subclade A comes as a surprise. Despite its lack of chalk glands, this white‐flowered species firstly assigned to section *Porphyrion* (which always has these structures but very many of whose species have white flowers). Zhang et al. (2015) moved it to sect. *Ciliatae* on account of its pollen morphology. *S. brevicaulis* was put in key 7 by Pan et al. (2001), corresponding to Gornall's subsect. *Serpyllifoliae*, but in our analyses it clusters at the base of subsect. *Hemisphaericae* and subsect. *Flagellares* with high support values. This species exhibits a cushion‐like habit and a setose‐ciliate apical margin of its basal leaves, somewhat similar to species of subsect. *Hemisphaericae*. Its close relatives, *S. sessiliflora* H.Sm. and *S. williamsii* H.Sm. (not studied here), may also belong here. Relationships among the taxa basal in subclade A need to be investigated further with improved taxonomic sampling but, for the moment, we prefer to retain subsect. *Hemisphaericae* as a convenient name under which to house these species.

The remaining part of subclade A comprises a monophyletic group of species with axillary, filiform stolons. These can be recognized as subsection *Flagellares* (Clarke) Engler and Irmscher (1912a), as in Gornall (1987) and in key 1 by Pan et al. (2001). We sampled five of the estimated 18 species of this subsection. With the exception of *S. brunonis*, sampled representatives of subsect. *Flagellares* form a clade highly supported as sister to *S*. *hemisphaerica*. The errant *S*. *brunonis*, despite possessing filiform stolons, occupies a position embedded among species of subsect. *Gemmiparae* in both this and in previous phylogenetic studies (Gao et al., 2015; Tkach et al., 2015). This suggests a parallel origin of axillary filiform stolons, a character that also occurs in the distantly related *S*. *stolonifera* Curtis of sect. *Irregulares* Haw. (Gao et al., 2015).

Pan (1991, 1992) divided *S*. *flaccida*, *S. nangxianensis*, *S. consanguinea*, *S. angustata* which we recognize as subsect. *Flagellares* into two subsections belonging to different sections, i.e. sect. *Ciliatae* subsect. *Flagellares* (with superior ovaries) and sect. *Ligulatae* subsect. *Microgynae* J‐T. Pan (with inferior ovaries). Of our sampled taxa in the present study, *S*. *flaccida* belongs to Pan's (1991, 1992) subsect. *Flagellares*, and the remaining three species (*S. nangxianensis*, *S. consanguinea*, *S. angustata*) represent Pan's (1991, 1992) subsect. *Microgynae*. The dramatic separation of these taxa into two sections is here shown to be unwarranted: support for an inclusive subsection *Flagellares* (minus *S. brunonis*) is high (PP = 1.00, BS = 100%, Figure 2).

Subclade B of clade 1 comprises species belonging to subsections *Gemmiparae* Engl. & Irmsch. and *Cinerascentes* Engl. & Irmsch. Eleven out of 20 species were sampled for this subsection, including a recently published species, *S*. *viridipetala* Z‐X. Zhang & Gornall (Gao et al., 2018). All representatives of subsect. *Gemmiparae* form a well‐supported monophyletic lineage in which *S*. *brunonis* and *S*. *cinerascens* are embedded. Species of subsect. *Gemmiparae* can be recognized by their leafy axillary buds (Engler & Irmscher, 1912a, 1912b, 1916/1919), although these can occasionally be missing. The filiform stolons in *S. brunonis* may be interpreted as developments of the leafy buds. The main distribution range of this subsection is the Himalaya‐Hengduan Mounatins region.

*Saxifraga cinerascens* is a narrowly distributed species restricted to north‐western Yunnan. It is the only species in subsection *Cinerascentes* and is recognized by its well‐developed leaf rosettes, cartilaginous setose‐ciliate leaf margin and aristate leaf apex (Engler & Irmscher, 1912a, 1912b, 1916/1919). Gornall (1987) initially accepted the subsection. Pan (1991, 1992), however, assigned the species to subsect. *Gemmiparae* Engl. & Irmsch., a position reaffirmed by Pan et al. (2001) who placed it in their key 2, which corresponds to subsect. *Gemmiparae*. A previous molecular phylogenetic study by Gao et al. (2015) also suggested that *S*. *cinerascens* is nested within subsect. *Gemmiparae*, a result supported by the present study. In fact, close examination of specimens shows that the species produces axillary leafy buds (diagnostic of the subsection) at proximal nodes, but these develop into sterile shoots by anthesis. Thus, it is convenient to abandon subsect. *Cinerascentes* and put *S*. *cinerascens* in subsect. *Gemmiparae*, based on molecular and morphological data.

Pan (1991, 1992) recognized four series within subsect. *Gemmiparae*, based on sepal and petal nervature and the presence and number of calloses. At least two (ser. *Brachypodae* C‐Y.Wu & J‐T.Pan and ser. *Dentosifoliae* J‐T.Pan) are not monophyletic; one is monotypic (ser. *Erinaceae* J‐T.Pan, with *S. erinacea* H.Sm.) and sampling is insufficient to comment on the other one (ser. *Spinulosae* (Clarke) Gornall). The two series recognized by Gornall (1987) correspond quite closely, but not exactly, to the clustering result of subclade B recovered in the present study (Figure 2). The basal position of *S. hispidula* D.Don renders one of them (ser. *Gemmiparae*) paraphyletic. The other (ser. *Spinulosae* (Clarke) Gornall) may well be monophyletic if *S. cinerascens* is included. But we do not propose adopting them at present, owing to the basal position of *S. hispidula*.

#### 4.3.2 Clade 2

This well‐supported clade consists of all species belonging to subsections *Rosulares* Gornall and *Serpyllifoliae* Gornall. We sampled 25 out of ca. 60 species of this group. This clade corresponds to Engler and Irmscher's (1916/1919) grex *Sediformes* Engl. & Irmsch., a later homonym of *Saxifraga* group *Sediformes* Reichenbach (1832) and therefore illegitimate. Gornall (1987) split this group into two subsections, subsect. *Rosulares* and subsect. *Serpyllifoliae*, based on whether a well‐defined basal leaf rosette was produced or not. From a habit viewpoint, species of subsect. *Rosulares* exhibit a cespitose habit, while plants of subsect. *Serpyllifoliae* are more mat‐like. Our plastome data presented in this study indicate that Engler and Irmcher's original taxon is monophyletic, and corresponds quite closely to Pan's (1991, 1992) delimitation of subsect *Rosulares*. Thus, the division into two subsections by Gornall (1987) is not supported by this study and the two are best amalgamated. Its name should be subsect. *Rosulares* Gornall, as selected by Pan (1991). The morphological synapomorphy of this subsection might be leaf rosettes either at the base of plants or terminal on sterile shoots.

Pan (1991, 1992) divided the clade into six series, but comparison of his lists of included species against the phylogenetic tree (Figure 2) shows that none of his series is monophyletic. The lumping approach adopted by Pan (1991, 1992) to the treatment of *S. umbellulata*, Hook.f. & Thoms., in which three varieties were recognized, viz. var. *umbellulata*, var. *pectinata* (Marqund & Airy Shaw) J‐T.Pan and var. *muricola* (Marqund & Airy Shaw) J‐T.Pan, is not supported by our data. Two of the varieties (var. *umbellulata* and var. *pectinata*) are well separated on the tree (Figure 2), supporting their recognition as separate species, *S. umbellulata* and *S. pasumensis* Marqand & Airy Shaw, respectively. The third variety was not studied.

#### 4.3.3 Clade 3

The third main clade recovered in our study, which is sister to clade 2, corresponds to subsect. *Hirculoideae* Engler and Irmscher (1912a), as delimited by Gornall (1987). It is the most species rich subsection of sect. *Ciliatae*, comprising more than 100 species of which we sampled 50 taxa. This clade has been identified in previous phylogenetic studies (Gao et al., 2015; Tkach et al., 2015; Zhang et al., 2008). Two morphological synapomorphies can be recognized for this subsection: (i) brown‐crisped hairs present at least at the proximal nodes of the stem (Engler & Irmscher, 1912a, 1912b); (ii) trinucleate pollen (Zhang & Gornall, 2011). As was described by Gao et al. (2015), three sub‐groups can be identified on morphological grounds: (i) species with brown‐crisped hairs on the pedicels (corresponds to key 3 of Pan et al., 2001); (ii) species with leaves having a glaucous abaxial surface and a prominent submarginal vein (corresponds to key 5 of Pan et al., 2001); and (iii) the remainder (corresponds to key 6 of Pan et al., 2001). In this study, our phylogenetic results indeed reveal three well‐supported subclades, but they correspond only partially with the morphological groups. Species with the three above‐mentioned characters are to some extent mixed on the phylogenetic tree.

Pan (1991, 1992) recognized six series and four subseries to accommodate the species, based on sepal vestiture, sepal and petal nervature and presence and number of calloses. None of them is monophyletic when compared to our tree (Figure 3), apart from two for which it is not possible to tell based on the current taxonomic sampling. Further morphological and micro‐morphological studies combining phylogenetic results with a wider sampling are needed to reveal species relationships of this species rich subsection.

It is worth mentioning that this paper reconstructs the phylogenetic relationships of sect. *Ciliatae* merely based on chloroplast data. Since chloroplast prone to capture following hybridization, conclusions based on chloroplast data alone do not represent the complete evolutionary history. Plastid capture has been reported across numerous plant lineages (Acosta & Premoli, 2010; Fehrer et al., 2007; Gurushidze et al., 2010; Mir et al., 2010; Okuyama et al., 2005). Although only few hybridizations have been recorded in this taxon, this does not mean that hybridization has never occurred, as well as influencing the evolutionary history. We will discuss the possible conflicting phenomena between nuclear and plastid phylogenies in the future research.

### 4.4 Taxonomic conclusions

The plastome phylogenetic results of sect. *Ciliatae* mostly agree with the classification of Gornall (1987), but some minor modifications should be made: (i) subsection *Hemisphaericae* requires further investigation, with better taxon sampling to establish its phylogenetic status and limits; (ii) Subsection *Gemmiparae* should include subsect. *Cinerascentes* (*S*. *cinerascens*); (iii) subsections *Rosulares* and *Serpyllifoliae* should be merged into one large subsection, for which the correct name is subsect. *Rosulares* Gornall. Section *Ciliatae* Haworth thus comprises at least five subsections as follows.

***Saxifraga* section *Ciliatae*** Haworth

1. **subsection *Gemmiparae*** Engler & Irmscher, *Notes R*. *Bot*. *Gard*. *Edinb*., *5*(*24*): 140 (1912).
  grex *Gemmiparae* (Engler & Irmscher) Engler & Irmscher, *Engl*. *bot*. *Jahrb*., *48*: 590 (1912).
  subsection *Cinerascentes* Engler & Irmscher, *Notes R*. *bot*. *Gard*. *Edinb*., *5*(*24*): 142 (1912).
  grex *Cinerascentes* (Engler & Irmscher) Engler & Irmscher, *Engl*. *bot*. *Jahrb*., *48*: 593 (1912).
2. **subsection *Hemisphaericae*** (Engler & Irmscher) Gornall, *Bot. J. Linn. Soc*., 95: 277 (1987). This subsection requires further investigation to establish its limits and phylogenetic status.
3. **subsection *Flagellares*** (C.B. Clarke) Engler & Irmscher, *Notes R*. *bot*. *Gard*. *Edinb*., *5*(*24*): 146 (1912).
4. **subsection *Rosulares*** Gornall, *Bot. J. Linn. Soc*., 95: 277 (1987).subsection *Sediformes* Engler & Irmscher, *Notes R*. *bot*. *Gard*. *Edinb*., *5*(*24*): 143 (1912), *nom. Illeg*., *non* group *Sediformes* H.G.L. Reichenbach (1832).
  grex *Sediformes* (Engler & Irmscher) Engler & Irmscher, *Engl*. *bot*. *Jahrb*., *48*: 594 (1912), *nom. illeg*.
  cycle *Sediformes* (Engler & Irmscher) Losina‐Losinskaya, *Fl. U.R.S.S*., *9*: 163 (1939), *nom. illeg*.
  subsection *Serpyllifoliae* Gornall, *Bot. J. Linn. Soc*., 95: 277 (1987).
5. **subsection *Hirculoideae*** Engler & Irmscher, *Notes R*. *bot*. *Gard*. *Edinb*., *5*(*24*): 135 (1912).

## 5 CONCLUSIONS

In this study, we successfully sequenced the complete chloroplast genome of 103 taxa, including 97 of *Saxifraga* and six of *Micranthes*. Combined with the NCBI database, the plastid genome data of 94 taxa of sect. *Ciliatae* were obtained. It is, up to now, the largest plastome data matrix concerning sect. *Ciliatae*. Chloroplast genomes of sect. *Ciliatae* are rather conservative in structural organization, gene arrangement, and gene content. Large structural variations, such as the deletion of IR regions, inversion of large fragments, and gene transposition are not detected in plastomes of sect. *Ciliatae*. However, gene loss and changes of IR boundaries were detected but with extremely low frequency, suggesting a conserved evolutionary pattern. Size variation of chloroplast genomes of sect. *Ciliatae* is probably caused by expansion/contraction of the IR regions. Eighty‐one PCGs shared by all taxa of sect. *Ciliatae* and outgroups were extracted for phylogenetic analyses, as well as the complete chloroplast genome sequences. The molecular phylogenetic trees exhibit high resolution and support values and confirm that sect. *Ciliatae* is monophyletic. Three well‐supported clades are revealed within sect. *Ciliatae*. Based on our phylogenetic results, different taxonomic systems of sect. *Ciliatae* are discussed and compared. Our phylogenetic results agree relatively well with subsections of Gornall (1987) but suggesting two minor modifications: (i) abandon the monotypic subsection *S*. subsect. *Cinerascentes* and put *S*. *cinerascens* in *S*. subsect. *Gemmiparae*; (ii) merge *S*. subsect. *Rosulares* and *S*. subsect. *Serpyllifoliae* one large subsection, *S*. subsect. *Sediformes*.

## AUTHOR CONTRIBUTIONS

**Rui Yuan:** Data curation (lead); formal analysis (lead); methodology (lead); software (lead); validation (lead); visualization (lead); writing – original draft (lead). **Xiaolei Ma:** Investigation (equal). **Zhouxin Zhang:** Writing – review and editing (equal). **Richard J. Gornall:** Writing – review and editing (equal). **Yongcui Wang:** Supervision (supporting). **Shilong Chen:** Supervision (equal). **Qingbo Gao:** Resources (lead); writing – review and editing (lead).

## CONFLICT OF INTEREST

None declared.

## Supporting information


Figure S1.

Figure S2.

Figure S3.

Figure S4.

Figure S5.

Figure S6.

Figure S7.

Figure S8.

Table S1.

Table S2.

## Data Availability

DNA sequences: Genbank accessions ON720850–ON720952.
