# Supplementary material for: Chloroplast phylogenomics and the taxonomy of Saxifraga section Ciliatae (Saxifragaceae)
Source: Ecol Evol. 2023 Jan 6;13(1):e9694. doi: 10.1002/ece3.9694 (PMC9817205; doi:10.1002/ece3.9694)
Supplement: Supplementary file 4 — Figure S4. [file ECE3-13-e9694-s010.docx]

Chloroplast phylogenomics and the taxonomy of *Saxifraga* section *Ciliatae* (Saxifragaceae)

Rui Yuan, Xiaolei Ma, Zhuoxin Zhang, Richard J. Gornall, Yongcui Wang, Shilong Chen, Qingbo Gao

**Appendix Figure S4** The nucleotide variability value (pi) in the chloroplast genome of *S.* sect. *Ciliatae*. (A) Protein-coding genes. (B) Intergenic regions.
