# Supplementary material for: Chloroplast phylogenomics and the taxonomy of Saxifraga section Ciliatae (Saxifragaceae)
Source: Ecol Evol. 2023 Jan 6;13(1):e9694. doi: 10.1002/ece3.9694 (PMC9817205; doi:10.1002/ece3.9694)
Supplement: Supplementary file 7 — Figure S7. [file ECE3-13-e9694-s005.docx]

Chloroplast phylogenomics and the taxonomy of *Saxifraga* section *Ciliatae* (Saxifragaceae)

Rui Yuan, Xiaolei Ma, Zhuoxin Zhang, Richard J. Gornall, Yongcui Wang, Shilong Chen, Qingbo Gao

(a)

(b)

**Appendix Figure S7** Phylogenetic tree reconstructed based on the CDS of the 81 protein-coding genes. (a) The tree uses Bayesian interference (BI) and maximum likelihood (ML) methods. Taxa include *S.* sect. *Ciliatae*, *Micranthes*, and outgroups in this study, but except *S*. subsect. *Hirculoideae.* Numbers at the nodes represent BI posterior probability (PP) and ML bootstrap (BS) values greater than 50%. The taxonomic information of *S.* sect. *Ciliatae* in previous studies and this study is arranged in columns. (b) This sub-figure is followed by (a). Based on the CDS from *S*. subsect. *Hirculoideae*, the phylogenetic tree was reconstructed by Bayesian interference (left) and maximum likelihood (right) methods. Numbers at the nodes represent BI posterior probability (PP) and ML bootstrap (BS) values greater than 50%. The taxonomic information of *S*. subsect. *Hirculoideae* is arranged in columns according to right tree.
