# Supplementary material for: Chloroplast phylogenomics and the taxonomy of Saxifraga section Ciliatae (Saxifragaceae)
Source: Ecol Evol. 2023 Jan 6;13(1):e9694. doi: 10.1002/ece3.9694 (PMC9817205; doi:10.1002/ece3.9694)
Supplement: Supplementary file 8 — Figure S8. [file ECE3-13-e9694-s003.docx]

Chloroplast phylogenomics and the taxonomy of *Saxifraga* section *Ciliatae* (Saxifragaceae)

Rui Yuan, Xiaolei Ma, Zhuoxin Zhang, Richard J. Gornall, Yongcui Wang, Shilong Chen, Qingbo Gao

(Manuscript ID: ECE-2022-09-01341)

(a)

(b)

(c)

(d)

(e)

(f)

**Appendix Figure S8** Gene species tree and five gene trees of the 122 taxa. (a) Gene species tree. (b) *rpoB* gene tree. (c) *rpoC2* gene tree. (d) *ndhF* gene tree. (e) *matK* gene tree. (f) *ycf1* gene tree.
