# Supplementary material for: Chloroplast phylogenomics and the taxonomy of Saxifraga section Ciliatae (Saxifragaceae)
Source: Ecol Evol. 2023 Jan 6;13(1):e9694. doi: 10.1002/ece3.9694 (PMC9817205; doi:10.1002/ece3.9694)
Supplement: Supplementary file 9 — Table S1. [file ECE3-13-e9694-s009.docx]

Chloroplast phylogenomics and the taxonomy of *Saxifraga* section *Ciliatae* (Saxifragaceae)

Rui Yuan, Xiaolei Ma, Zhuoxin Zhang, Richard J. Gornall, Yongcui Wang, Shilong Chen, Qingbo Gao

**Appendix Table S1** The origin of materials, including the information of taxon, locality voucher, and GenBank accession numbers

| **Taxon** | **Locality** | **VouCher** | **Genbank Acc. No.** |
| --- | --- | --- | --- |
| ***Saxifraga* L*.***  **sect. *Ciliatae* Haw.**  **subsect. *Hirculoideae* Engl. & Irmsch.** |  |  |  |
| *S. aristulata* J.D.Hook. & Thoms. | Ganzi, Sichuan | Chen2012223 | ON720851 |
| *S. aristulata var. longipila*  (Engl.et Irmsch.) J.T.Pan | Diqing, Yunnan | Chen2012143 | ON720852 |
| *S. auriculata* Engl. & Irmsch. | Aba, Sichuan | Chen06112 | ON720855 |
| *S. auriculata var. conaensis* J-T. Pan | Cuona, Xizang | Chen2014446-1 | ON720856 |
| *S. bergenioides* Marquand | Longzi, Xizang | Chen2014367-8 | ON720858 |
| *S. chumbiensis* Engl. & Irmsch. | Yadong, Xizang | Chen2014505-4 | ON720864 |
| *S. congestiflora* Engl. & Irmsch. | Dari, Qinghai | Gao2018173-1 | ON720866 |
| *S. diversifolia* Wall. ex Ser. | Yanyuan, Sichuan | Chen2013522 | ON720868 |
| *S. diversifolia var. angustibracteata*  (Engl.et Irmsch.) J.T.Pan | Muli, Sichuan | Chen03107 | ON720869 |
| *S. eglandulosa* Engl. | Cuona, Xizang | Chen2014461-4 | ON720871 |
| *S. egregia* Engl. | Seda, Sichuan | Gao2018170-1 | ON720872 |
| *S. egregia var. eciliata* Engl. | Aba, Sichuan | Chen03035-6 | ON720873 |
| *S. erectisepala* J-T. Pan | Yulong, Yunnan | Chen06154 | ON720874 |
| *S. glabricaulis* H. Sm. | Cuona, Xizang | Chen2014430 | ON720913 |
| *S. heleonastes* H. Sm. | Xinghai, Qinghai | Gao2018134-4 | ON720886 |
| *S. hirculoides* Decne. | Loza, Xizang | Chen2014484-2 | ON720888 |
| *S. hookeri* Engl. & Irmsch. | Gongbujiangda, Xizang | Chen2007128 | ON720890 |
| *S. hypericoides* Franch. | Deqin, Yunnan | Chen2012142-2 | ON720891 |
| *S. implicans* H. Sm. | Deqin, Yunnan | Chen2012137-1 | ON720892 |
| *S. insolens* Irmsch. | Shangrila, Yunnan | Chen2012115-3 | ON720893 |
| *S. isophylla* H. Sm. | Nyingchi, Xizang | Chen2014314-1 | ON720894 |
| *S. kingdonii* Marquand | Yadong, Xizang | Chen2013469 | ON720895 |
| *S. lepida* H. Sm. | Cuona, Xizang | Chen2014460 | ON720896 |
| *S. litangensis* Engl. | Leiwuqi, Xizang | Gao2018151-9 | ON720897 |
| *S. lychnitis* J.D.Hook. & Thoms. | Leiwuqi, Xizang | Gao2018152-4 | ON720898 |
| *S. maxionggouensis* J-T. Pan | Daocheng, Sichuan | Chen2012218-4 | ON720900 |
| *S. montanella* H. Sm. | Loza, Xizang | Chen2014483-6 | ON720905 |
| *S. moorcroftiana* (Wall. ex Ser.) Sternb. | Yadong, Xizang | Chen2014510-2 | ON720906 |
| *S. nangqenica* J-T. Pan | Xinghai, Qinghai | Gao2018119-1 | ON720909 |
| *S. nigroglandulifera* Balakr. | Daocheng, Sichuan | Chen2012196-1 | ON720911 |
| *S. oresbia* Anth. | Litang, Sichuan | Chen2012239-4 | ON720912 |
| *S. pardanthina* Hand.-Mazz. | Xiangcheng, Sichuan | Chen06243 | ON720914 |
| *S. parnassiifolia* D. Don | Cuona, Xizang | Chen2014427-2 | ON720915 |
| *S. parva* Hemsl. | Maduo, Qinghai | Chensl2011018 | ON720916 |
| *S. pratensis* Engl. & Irmsch. | Zhongdian, Yunnan | Chen06199 | ON720918 |
| *S. przewalskii* Engl. | Qilian, Qinghai | Gao2017011-4 | ON720919 |
| *S. pseudohirculus* Engl. | Seda, Sichuan | Gao2018167-1 | ON720920 |
| *S. saginoides* J.D.Hook. & Thoms. | Longzi, Xizang | Chen2014368-5 | ON720923 |
| *S. sikkimensis* Engl. | Cuona, Xizang | Chen2014447-2 | ON720928 |
| *S. sinomontana* J-T. Pan & Gornall |  | from GenBank | MN104589 |
| *S. sinomontana var. amabilis* |  |  |  |
| H. Sm. ex J-T. Pan | Deqin, Yunnan | Chen03171 | ON720929 |
| *S. stellariifolia* Franch. | Aba, Sichuan | Chen06089 | ON720931 |
| *S. subaequifoliata* Irmsch. | Longzi, Xizang | Chen2013265 | ON720932 |
| *S. tangutica* Engl. | Xinghai, Qinghai | Gao2018115-2 | ON720934 |
| *S. tangutica var. platyphylla* |  |  |  |
| (H.Smith) J.T.Pan | Ganzi, Sichuan | Chen2014210-5 | ON720935 |
| *S. tibetica* A. Los. | Yushu, Qinghai | Gao2018123-4 | ON720937 |
| *S. tsangchanensis* Franch. | Cuona, Xizang | Chen2013330 | ON720938 |
| *S. viscidula* J.D.Hook. & Thoms. | Yadong, Xizang | Chen2014511-2 | ON720947 |
| *S. xiaozhongdianensis* J-T. Pan | Zhongdian, Yunnan | Chen03168 | ON720950 |
| *S. yarlungzangboensis* J-T. Pan | Cuona, Xizang | Chen2014456 | ON720951 |
| **subsect. *Flagellares* (C. B. Clarke) Engler & Irmscher** | | |  |
| *S. angustata* H. Sm. | Diqing, Yunnan | Chen2012140-3 | ON720850 |
| *S. brunonis* Wall. ex Ser. | Cuona, Xizang | Chen2014448 | ON720862 |
| *S. consanguinea* W.W.Sm. | Xinghai, Qinghai | Gao2018118-1 | ON720867 |
| *S. flaccida* J-T. Pan | Yadong, Xizang | Chen2013466-2 | ON720876 |
| *S. nangxianensis* J-T. Pan | Cuona, Xizang | Chen2014410-4 | ON720910 |
| **subsect. *Rosulares* Gornall** |  |  |  |
| *S. atuntsiensis* W.W.Sm. | Yushu, Qinghai | Chen2014542-1 | ON720853 |
| *S. brunneopunctata* H. Sm. | Maizhokunggar, Xizang | Chen2007122 | ON720861 |
| *S. gyalana* Marquand & Airy-Shaw | Changdu, Xizang | Gao2018154-4 | ON720885 |
| *S. punctulata* Engl. | Naidong, Xizang | Chen2014358-1 | ON720921 |
| *S. sanguinea* Franch. | Shiqu, Sichuan | Chen2014234-5 | ON720924 |
| *S. sediformis* Engl. & Irmsch. | Yulong, Yunnan | Chen2012104-2 | ON720925 |
| *S. signata* Engl. & Irmsch. | Jiangda, Xizang | Chen2012457-1 | ON720926 |
| *S. signatella* Marquand | Longzi, Xizang | Chen2014380-7 | ON720927 |
| *S. taraktophylla* Marquand & Airy-Shaw | Cuomei, Xizang | Chen2014478-1 | ON720936 |
| *S. umbellulata* J.D.Hook. & Thoms. | Longzi, Xizang | Chen2013298 | ON720939 |
| *S. umbellulata var. pectinata* |  |  |  |
| (Marquand & Airy-Shaw) J-T. Pan | Nyingchi, Xizang | Chen2014311-5 | ON720940 |
| *S. unguiculata* Engl. | Xinghai, Qinghai | Gao2018117-1 | ON720941 |
| *S. unguiculata var. limprichtii* |  |  |  |
| (Engl. & Irmsch.) J-T. Pan | Xunhua, Qinghai | Chen2014003-2 | ON720942 |
| *S. unguiculata var. subglabra* Engl. | Bama, Qinghai | Chen2014145-1 | ON720943 |
| *S. vilmoriniana* Engl. & Irmsch. | Changdu, Xizang | Chen2014283-8 | ON720945 |
| *S. yushuensis* J-T. Pan | Yushu, Qinghai | Gao2018121-5 | ON720952 |
| **subsect. *Serpyllifoliae* Gornall** |  |  |  |
| *S. aurantiaca* Franch. | Aba, Sichuan | Chen2012047-5 | ON720854 |
| *S. brevicaulis* H. Sm. | Cuona, Xizang | Chen2014411-3 | ON720860 |
| *S. drabiformis* Franch. | Yulong, Yunnan | Chen06194 | ON720870 |
| *S. gemmigera* Engl. | Dari, Qinghai | Chensl2011010 | ON720878 |
| *S. gemmigera var. gemmuligera* |  |  |  |
| (Engler) J-T. Pan & Gornall | Dari, Qinghai | Gao2018175-1 | ON720879 |
| *S. glacialis* H. Sm. | Seda, Sichuan | Chen2014165-3 | ON720881 |
| *S. nanella* Engl. & Irmsch. | Yushu, Qinghai | Gao2018124-3 | ON720908 |
| *S. perpusilla* J.D.Hook. & Thoms. | Longzi, Xizang | Chen2014389-1 | ON720917 |
| *S. stella-aurea* J.D.Hook. & Thoms. | Yushu, Qinghai | Chen2014540-3 | ON720930 |
| *S. uninervia* Anthony | Cuona, Xizang | Chen2014413 | ON720944 |
| **subsect. *Gemmiparae* Engl. & Irmsch.** |  |  |  |
| *S. balfourii* Engl. & Irmsch. | Lijiang, Yunnan | Chen2012076-5 | ON720857 |
| *S. brachypoda* D. Don | Yadong, Xizang | Chen2014523 | ON720859 |
| *S. filicaulis* Wall. ex Ser. | Cuona, Xizang | Chen2014425-1 | ON720875 |
| *S. gemmipara* Franch. | Yanyuan, Sichuan | Chen2013529 | ON720880 |
| *S. gouldii* C. E. C. Fischer | Yadong, Xizang | Chen2014509-1 | ON720882 |
| *S. gouldii var. eglandulosa* H. Sm. | Cuona, Xizang | Chen2014449-3 | ON720883 |
| *S. hispidula* D. Don | Cuona, Xizang | Chen2014450-5 | ON720889 |
| *S. substrigosa* J-T. Pan | Nyingchi, Xizang | Chen2014304-3 | ON720933 |
| *S. viridipetala* Z. X. Zhang & Gornall | Huili, Sichuan | Gao2015006-1 | ON720946 |
| *S. wallichiana* Sternb. | Yadong, Xizang | Chen2014507-3 | ON720948 |
| *S. wardii* W.W.Sm. | Nyingchi, Xizang | Chen2014313-1 | ON720949 |
| **subsect.** ***Cinerascentes* Engler & Irmscher** |  |  |  |
| *S. cinerascens* Engl. & Irmsch. | Yulong, Yunnan | Chen2012078-4 | ON720865 |
| **subsect. *Hemisphaericae* (Engl. & Irmsch.) Gornall** | |  |  |
| *S. hemisphaerica* J.D.Hook. & Thoms. | Yushu, Qinghai | Gao2018125-4 | ON720887 |
| **sect. *Mesogyne* Sternb.** |  |  |  |
| *S. cernua* L. | Qilian, Qinghai | Gao2017009-2 | ON720863 |
| *S. granulifera* H. Sm. | Bama, Qinghai | Chen2014146 | ON720884 |
| **sect. *Irregulares* Haw.** |  |  |  |
| *S. fortunei* Hook. f. | Yanyuan, Sichuan | Chen2013526 | ON720877 |
| *S. rufescens* Balf. f. | Yulong, Yunnan | Chen2012077-4 | ON720922 |
| *S. stolonifera* Curt. |  | from GenBank | MN496079 |
| ***Micranthes* Haw.** |  |  |  |
| *Micranthes. atrata* (Engl.) Losinsk. | Tianjun, Qinghai | Gao2020217-20 | ON720899 |
| *Micranthes. davidii* (Franch.) Losinsk. | Dari, Qinghai | Chensl2011012 | ON720901 |
| *Micranthes. divaricata*  (Engl. & Irmsch.) Losinsk. | Daocheng, Sichuan | Chen2012219-1 | ON720902 |
| *Micranthes. melanocentra* (Franch.) Losinsk. | Dege, Sichuan | Gao2018157-3 | ON720903 |
| *Micranthes. melanocentra var. rubriflora*  J-T. Pan | Daocheng, Sichuan | Chen2012192 | ON720904 |
| *Micranthes. pallida* (Wall. ex Ser.) Losinsk. | Cuona, Xizang | Chen2014429-3 | ON720907 |
| ***Chrysosplenium* Tourn. ex L.** |  |  |  |
| *Chrysosplenium. flagelliferum* Fr. Schmidt. |  | from GenBank | MN729584 |
| *Chrysosplenium. macrophyllum* Oliv. |  | from GenBank | MK973001 |
| *Chrysosplenium. lanuginosum*  J.D.Hook. & Thoms. |  | from GenBank | MK814607 |
| ***Heuchera* L.** |  |  |  |
| *Heuchera. abramsii* Rydb. |  | from GenBank | MN496062 |
| *Heuchera. alba* Rydb. |  | from GenBank | MN496063 |
| ***Tanakaea* Franch. & Sav.** |  |  |  |
| *Tanakaea. radicans* Franchet & Savatier |  | from GenBank | NC_057123 |
| ***Tiarella* L.** |  |  |  |
| *Tiarella. trifoliata* L. |  | from GenBank | NC_042929 |
| ***Mitella* Tourn. ex L.** |  |  |  |
| *Mitella. formosana* (Hayata) Masam. |  | from GenBank | NC_042926 |
| ***Leptarrhena* R. Br.** |  |  |  |
| *Leptarrhena. pyrolifolia* (D.Don) Ser. |  | from GenBank | MN496070 |
| ***Mukdenia* Koidz.** |  |  |  |
| *Mukdenia. rossii* (Oliv.) Koidz. |  | from GenBank | NC_037495 |
| ***Oresitrophe* Bunge** |  |  |  |
| *Oresitrophe. rupifraga* Bunge |  | from GenBank | MN496074 |
| ***Rodgersia* A. Gray** |  |  |  |
| *Rodgersia. sambucifolia* Hemsl. |  | from GenBank | MN496077 |
| ***Ribes* L.** |  |  |  |
| *Ribes. fasciculatum var. chinense* Maxim. |  | from GenBank | MH191388 |
| *Ribes. nevadense* Kellogg |  | from GenBank | MN496075 |
| *Ribes. odoratum* Wendland |  | from GenBank | MT081309 |
| *Ribes. roezlii* Regel |  | from GenBank | MN496076 |
| ***Itea* L.** |  |  |  |
| *Itea. chinensis* Hook. & Arn. |  | from GenBank | NC_037884 |
