# Supplementary material for: Chloroplast phylogenomics and the taxonomy of Saxifraga section Ciliatae (Saxifragaceae)
Source: Ecol Evol. 2023 Jan 6;13(1):e9694. doi: 10.1002/ece3.9694 (PMC9817205; doi:10.1002/ece3.9694)
Supplement: Supplementary file 10 — Table S2. [file ECE3-13-e9694-s004.docx]

Chloroplast phylogenomics and the taxonomy of *Saxifraga* section *Ciliatae* (Saxifragaceae)

Rui Yuan, Xiaolei Ma, Zhuoxin Zhang, Richard J. Gornall, Yongcui Wang, Shilong Chen, Qingbo Gao

**Appendix Table S2** Types, numbers and percentages of SSRs in the chloroplast genomes of *S.* sect. *Ciliatae*

| Taxon | p1 | | p2 | | p3 | | p4 | | p5 | | p6 | | Totall |
| --- | --- | --- | --- | --- | --- | --- | --- | --- | --- | --- | --- | --- | --- |
|  | No./% | | No./% | | No./% | | No./% | | No./% | | No./% | | No. |
| *S. angustata* | 54 | 65.85 | 5 | 6.10 | 11 | 13.41 | 5 | 6.10 | 1 | 1.22 | 6 | 7.32 | 82 |
| *S. aristulata* | 48 | 82.76 | 3 | 5.17 | 3 | 5.17 | 4 | 6.90 | 0 | 0.00 | 0 | 0.00 | 58 |
| *S. aristulata var longipila* | 42 | 79.25 | 4 | 7.55 | 3 | 5.66 | 4 | 7.55 | 0 | 0.00 | 0 | 0.00 | 53 |
| *S. atuntsiensis* | 48 | 78.69 | 3 | 4.92 | 3 | 4.92 | 5 | 8.20 | 1 | 1.64 | 1 | 1.64 | 61 |
| *S. aurantiaca* | 51 | 83.61 | 3 | 4.92 | 2 | 3.28 | 5 | 8.20 | 0 | 0.00 | 0 | 0.00 | 61 |
| *S. auriculata* | 47 | 82.46 | 3 | 5.26 | 3 | 5.26 | 4 | 7.02 | 0 | 0.00 | 0 | 0.00 | 57 |
| *S. auriculata var conaensis* | 46 | 80.70 | 4 | 7.02 | 2 | 3.51 | 4 | 7.02 | 1 | 1.75 | 0 | 0.00 | 57 |
| *S. balfourii* | 44 | 70.97 | 4 | 6.45 | 4 | 6.45 | 8 | 12.90 | 0 | 0.00 | 2 | 3.23 | 62 |
| *S. bergenioides* | 59 | 80.82 | 4 | 5.48 | 4 | 5.48 | 4 | 5.48 | 2 | 2.74 | 0 | 0.00 | 73 |
| *S. brachypoda* | 43 | 71.67 | 4 | 6.67 | 4 | 6.67 | 7 | 11.67 | 0 | 0.00 | 2 | 3.33 | 60 |
| *S. brevicaulis* | 55 | 80.88 | 3 | 4.41 | 3 | 4.41 | 6 | 8.82 | 1 | 1.47 | 0 | 0.00 | 68 |
| *S. brunneopunctata* | 44 | 81.48 | 5 | 9.26 | 4 | 7.41 | 0 | 0.00 | 0 | 0.00 | 1 | 1.85 | 54 |
| *S. brunonis* | 57 | 83.82 | 2 | 2.94 | 2 | 2.94 | 7 | 10.29 | 0 | 0.00 | 0 | 0.00 | 68 |
| *S. chumbiensis* | 49 | 73.13 | 7 | 10.45 | 3 | 4.48 | 4 | 5.97 | 2 | 2.99 | 2 | 2.99 | 67 |
| *S. cinerascens* | 50 | 75.76 | 3 | 4.55 | 4 | 6.06 | 8 | 12.12 | 1 | 1.52 | 0 | 0.00 | 66 |
| *S. congestiflora* | 48 | 81.36 | 2 | 3.39 | 3 | 5.08 | 4 | 6.78 | 1 | 1.69 | 1 | 1.69 | 59 |
| *S. consanguinea* | 51 | 68.00 | 5 | 6.67 | 8 | 10.67 | 6 | 8.00 | 1 | 1.33 | 4 | 5.33 | 75 |
| *S. diversifolia* | 42 | 72.41 | 3 | 5.17 | 7 | 12.07 | 5 | 8.62 | 1 | 1.72 | 0 | 0.00 | 58 |
| *S. diversifolia var angustibracteata* | 43 | 84.31 | 2 | 3.92 | 2 | 3.92 | 4 | 7.84 | 0 | 0.00 | 0 | 0.00 | 51 |
| *S. drabiformis* | 55 | 82.09 | 3 | 4.48 | 3 | 4.48 | 5 | 7.46 | 0 | 0.00 | 1 | 1.49 | 67 |
| *S. eglandulosa* | 38 | 76.00 | 5 | 10.00 | 2 | 4.00 | 3 | 6.00 | 2 | 4.00 | 0 | 0.00 | 50 |
| *S. egregia* | 43 | 81.13 | 4 | 7.55 | 2 | 3.77 | 4 | 7.55 | 0 | 0.00 | 0 | 0.00 | 53 |
| *S. egregia var eciliata* | 45 | 83.33 | 3 | 5.56 | 2 | 3.70 | 4 | 7.41 | 0 | 0.00 | 0 | 0.00 | 54 |
| *S. erectisepala* | 47 | 82.46 | 4 | 7.02 | 2 | 3.51 | 4 | 7.02 | 0 | 0.00 | 0 | 0.00 | 57 |
| *S. filicaulis* | 43 | 71.67 | 4 | 6.67 | 4 | 6.67 | 7 | 11.67 | 0 | 0.00 | 2 | 3.33 | 60 |
| *S. flaccida* | 46 | 69.70 | 5 | 7.58 | 7 | 10.61 | 5 | 7.58 | 1 | 1.52 | 2 | 3.03 | 66 |
| *S. gemmigera* | 43 | 75.44 | 4 | 7.02 | 5 | 8.77 | 5 | 8.77 | 0 | 0.00 | 0 | 0.00 | 57 |
| *S. gemmigera var gemmuligera* | 43 | 75.44 | 4 | 7.02 | 5 | 8.77 | 5 | 8.77 | 0 | 0.00 | 0 | 0.00 | 57 |
| *S. gemmipara* | 48 | 77.42 | 4 | 6.45 | 3 | 4.84 | 5 | 8.06 | 0 | 0.00 | 2 | 3.23 | 62 |
| *S. glacialis* | 46 | 80.70 | 3 | 5.26 | 3 | 5.26 | 5 | 8.77 | 0 | 0.00 | 0 | 0.00 | 57 |
| *S. gouldii* | 45 | 81.82 | 1 | 1.82 | 4 | 7.27 | 5 | 9.09 | 0 | 0.00 | 0 | 0.00 | 55 |
| *S. gouldi var eglandulosa* | 50 | 78.13 | 3 | 4.69 | 4 | 6.25 | 7 | 10.94 | 0 | 0.00 | 0 | 0.00 | 64 |
| *S. gyalana* | 44 | 77.19 | 5 | 8.77 | 3 | 5.26 | 5 | 8.77 | 0 | 0.00 | 0 | 0.00 | 57 |
| *S. heleonastes* | 40 | 81.63 | 2 | 4.08 | 3 | 6.12 | 3 | 6.12 | 1 | 2.04 | 0 | 0.00 | 49 |
| *S. hemisphaerica* | 60 | 81.08 | 5 | 6.76 | 5 | 6.76 | 4 | 5.41 | 0 | 0.00 | 0 | 0.00 | 74 |
| *S. hirculoides* | 47 | 79.66 | 4 | 6.78 | 3 | 5.08 | 4 | 6.78 | 1 | 1.69 | 0 | 0.00 | 59 |
| *S. hispidula* | 56 | 83.58 | 3 | 4.48 | 2 | 2.99 | 6 | 8.96 | 0 | 0.00 | 0 | 0.00 | 67 |
| *S. hookeri* | 48 | 76.19 | 5 | 7.94 | 4 | 6.35 | 4 | 6.35 | 2 | 3.17 | 0 | 0.00 | 63 |
| *S. hypericoides* | 40 | 78.43 | 3 | 5.88 | 4 | 7.84 | 4 | 7.84 | 0 | 0.00 | 0 | 0.00 | 51 |
| *S. implicans* | 44 | 78.57 | 4 | 7.14 | 3 | 5.36 | 5 | 8.93 | 0 | 0.00 | 0 | 0.00 | 56 |
| *S. insolens* | 47 | 77.05 | 7 | 11.48 | 3 | 4.92 | 3 | 4.92 | 1 | 1.64 | 0 | 0.00 | 61 |
| *S. isophylla* | 41 | 77.36 | 4 | 7.55 | 4 | 7.55 | 4 | 7.55 | 0 | 0.00 | 0 | 0.00 | 53 |
| *S. kingdonii* | 45 | 78.95 | 4 | 7.02 | 4 | 7.02 | 3 | 5.26 | 1 | 1.75 | 0 | 0.00 | 57 |
| *S. lepida* | 48 | 81.36 | 5 | 8.47 | 1 | 1.69 | 4 | 6.78 | 1 | 1.69 | 0 | 0.00 | 59 |
| *S. litangensis* | 43 | 79.63 | 4 | 7.41 | 3 | 5.56 | 4 | 7.41 | 0 | 0.00 | 0 | 0.00 | 54 |
| *S. lychnitis* | 46 | 82.14 | 2 | 3.57 | 3 | 5.36 | 4 | 7.14 | 1 | 1.79 | 0 | 0.00 | 56 |
| *S. maxionggouensis* | 47 | 85.45 | 3 | 5.45 | 3 | 5.45 | 2 | 3.64 | 0 | 0.00 | 0 | 0.00 | 55 |
| *S. montanella* | 53 | 76.81 | 3 | 4.35 | 4 | 5.80 | 5 | 7.25 | 4 | 5.80 | 0 | 0.00 | 69 |
| *S. moorcroftiana* | 42 | 76.36 | 5 | 9.09 | 1 | 1.82 | 5 | 9.09 | 1 | 1.82 | 1 | 1.82 | 55 |
| *S. nanella* | 50 | 84.75 | 3 | 5.08 | 3 | 5.08 | 3 | 5.08 | 0 | 0.00 | 0 | 0.00 | 59 |
| *S. nangqenica* | 49 | 81.67 | 3 | 5.00 | 3 | 5.00 | 4 | 6.67 | 1 | 1.67 | 0 | 0.00 | 60 |
| *S. nangxianensis* | 46 | 67.65 | 5 | 7.35 | 8 | 11.76 | 5 | 7.35 | 1 | 1.47 | 3 | 4.41 | 68 |
| *S. nigroglandulifera* | 50 | 75.76 | 7 | 10.61 | 4 | 6.06 | 5 | 7.58 | 0 | 0.00 | 0 | 0.00 | 66 |
| *S. oresbia* | 45 | 84.91 | 2 | 3.77 | 2 | 3.77 | 4 | 7.55 | 0 | 0.00 | 0 | 0.00 | 53 |
| *S. glabricaulis* | 47 | 82.46 | 2 | 3.51 | 3 | 5.26 | 4 | 7.02 | 1 | 1.75 | 0 | 0.00 | 57 |
| *S. pardanthina* | 44 | 81.48 | 4 | 7.41 | 2 | 3.70 | 4 | 7.41 | 0 | 0.00 | 0 | 0.00 | 54 |
| *S. parnassiifolia* | 40 | 72.73 | 7 | 12.73 | 2 | 3.64 | 5 | 9.09 | 1 | 1.82 | 0 | 0.00 | 55 |
| *S. parva* | 41 | 78.85 | 2 | 3.85 | 3 | 5.77 | 4 | 7.69 | 1 | 1.92 | 1 | 1.92 | 52 |
| *S. perpusilla* | 44 | 78.57 | 2 | 3.57 | 3 | 5.36 | 5 | 8.93 | 0 | 0.00 | 2 | 3.57 | 56 |
| *S. pratensis* | 45 | 80.36 | 5 | 8.93 | 2 | 3.57 | 4 | 7.14 | 0 | 0.00 | 0 | 0.00 | 56 |
| *S. przewalskii* | 42 | 80.77 | 2 | 3.85 | 3 | 5.77 | 5 | 9.62 | 0 | 0.00 | 0 | 0.00 | 52 |
| *S. pseudohirculus* | 40 | 75.47 | 5 | 9.43 | 4 | 7.55 | 4 | 7.55 | 0 | 0.00 | 0 | 0.00 | 53 |
| *S. punctulata* | 47 | 73.44 | 5 | 7.81 | 2 | 3.13 | 7 | 10.94 | 3 | 4.69 | 0 | 0.00 | 64 |
| *S. saginoides* | 38 | 77.55 | 4 | 8.16 | 2 | 4.08 | 4 | 8.16 | 1 | 2.04 | 0 | 0.00 | 49 |
| *S. sanguinea* | 42 | 82.35 | 3 | 5.88 | 2 | 3.92 | 4 | 7.84 | 0 | 0.00 | 0 | 0.00 | 51 |
| *S. sediformis* | 42 | 79.25 | 3 | 5.66 | 3 | 5.66 | 5 | 9.43 | 0 | 0.00 | 0 | 0.00 | 53 |
| *S. signata* | 41 | 78.85 | 2 | 3.85 | 3 | 5.77 | 6 | 11.54 | 0 | 0.00 | 0 | 0.00 | 52 |
| *S. signatella* | 46 | 80.70 | 2 | 3.51 | 2 | 3.51 | 5 | 8.77 | 0 | 0.00 | 2 | 3.51 | 57 |
| *S. sikkimensis* | 39 | 76.47 | 6 | 11.76 | 1 | 1.96 | 3 | 5.88 | 2 | 3.92 | 0 | 0.00 | 51 |
| *S. sinomontana* | 41 | 74.55 | 4 | 7.27 | 5 | 9.09 | 4 | 7.27 | 1 | 1.82 | 0 | 0.00 | 55 |
| *S. sinomontana var amabilis* | 44 | 81.48 | 1 | 1.85 | 3 | 5.56 | 4 | 7.41 | 2 | 3.70 | 0 | 0.00 | 54 |
| *S. stellaaurea* | 50 | 84.75 | 3 | 5.08 | 3 | 5.08 | 3 | 5.08 | 0 | 0.00 | 0 | 0.00 | 59 |
| *S. stellariifolia* | 41 | 78.85 | 4 | 7.69 | 3 | 5.77 | 4 | 7.69 | 0 | 0.00 | 0 | 0.00 | 52 |
| *S. subaequifoliata* | 43 | 78.18 | 4 | 7.27 | 4 | 7.27 | 4 | 7.27 | 0 | 0.00 | 0 | 0.00 | 55 |
| *S. substrigosa* | 43 | 72.88 | 4 | 6.78 | 4 | 6.78 | 6 | 10.17 | 0 | 0.00 | 2 | 3.39 | 59 |
| *S. tangutica* | 39 | 72.22 | 4 | 7.41 | 5 | 9.26 | 4 | 7.41 | 2 | 3.70 | 0 | 0.00 | 54 |
| *S. tangutica var platyphylla* | 43 | 79.63 | 4 | 7.41 | 2 | 3.70 | 4 | 7.41 | 1 | 1.85 | 0 | 0.00 | 54 |
| *S. taraktophylla* | 40 | 75.47 | 4 | 7.55 | 3 | 5.66 | 5 | 9.43 | 0 | 0.00 | 1 | 1.89 | 53 |
| *S. tibetica* | 41 | 77.36 | 4 | 7.55 | 4 | 7.55 | 4 | 7.55 | 0 | 0.00 | 0 | 0.00 | 53 |
| *S. tsangchanensis* | 48 | 72.73 | 8 | 12.12 | 3 | 4.55 | 4 | 6.06 | 2 | 3.03 | 1 | 1.52 | 66 |
| *S. umbellulata* | 46 | 82.14 | 3 | 5.36 | 3 | 5.36 | 4 | 7.14 | 0 | 0.00 | 0 | 0.00 | 56 |
| *S. umbellulata var pectinata* | 43 | 78.18 | 4 | 7.27 | 3 | 5.45 | 5 | 9.09 | 0 | 0.00 | 0 | 0.00 | 55 |
| *S. unguiculata* | 46 | 77.97 | 4 | 6.78 | 4 | 6.78 | 5 | 8.47 | 0 | 0.00 | 0 | 0.00 | 59 |
| *S. unguiculata var limprichtii* | 51 | 82.26 | 3 | 4.84 | 2 | 3.23 | 5 | 8.06 | 1 | 1.61 | 0 | 0.00 | 62 |
| *S. unguiculata var subglabra* | 42 | 76.36 | 4 | 7.27 | 5 | 9.09 | 4 | 7.27 | 0 | 0.00 | 0 | 0.00 | 55 |
| *S. uninervia* | 42 | 79.25 | 3 | 5.66 | 3 | 5.66 | 5 | 9.43 | 0 | 0.00 | 0 | 0.00 | 53 |
| *S. vilmoriniana* | 44 | 80.00 | 3 | 5.45 | 3 | 5.45 | 5 | 9.09 | 0 | 0.00 | 0 | 0.00 | 55 |
| *S. viridipetala* | 50 | 78.13 | 4 | 6.25 | 3 | 4.69 | 5 | 7.81 | 0 | 0.00 | 2 | 3.13 | 64 |
| *S. viscidula* | 41 | 77.36 | 5 | 9.43 | 3 | 5.66 | 4 | 7.55 | 0 | 0.00 | 0 | 0.00 | 53 |
| *S. wallichiana* | 44 | 73.33 | 3 | 5.00 | 4 | 6.67 | 6 | 10.00 | 2 | 3.33 | 1 | 1.67 | 60 |
| *S. wardii* | 49 | 79.03 | 3 | 4.84 | 4 | 6.45 | 6 | 9.68 | 0 | 0.00 | 0 | 0.00 | 62 |
| *S. xiaozhongdianensis* | 41 | 77.36 | 4 | 7.55 | 3 | 5.66 | 5 | 9.43 | 0 | 0.00 | 0 | 0.00 | 53 |
| *S. yarlungzangboensis* | 42 | 77.78 | 5 | 9.26 | 3 | 5.56 | 4 | 7.41 | 0 | 0.00 | 0 | 0.00 | 54 |
| *S. yushuensis* | 57 | 85.07 | 2 | 2.99 | 3 | 4.48 | 5 | 7.46 | 0 | 0.00 | 0 | 0.00 | 67 |
